# MTSS1 curtails lung adenocarcinoma immune evasion by promoting AIP4-mediated PD-L1 monoubiquitination and lysosomal degradation

**DOI:** 10.1038/s41421-022-00507-x

**Published:** 2023-02-21

**Authors:** Yuan Wang, Zhenchang Jia, Chenxi Liang, Yunfei He, Min Cong, Qiuyao Wu, Pu Tian, Dasa He, Xiang Miao, Beibei Sun, Yue Yin, Chao Peng, Feng Yao, Da Fu, Yajun Liang, Peiyuan Zhang, Hua Xiong, Guohong Hu

**Affiliations:** 1 Shanghai Institute of Nutrition and Health, University of Chinese Academy of Sciences, Chinese Academy of Sciences, Shanghai, China; 2 Department of Thoracic Surgery, Shanghai Chest Hospital, Shanghai Jiaotong University, Shanghai, China; 3 National Facility for Protein Science in Shanghai, Zhangjiang Lab, Shanghai Advanced Research Institute, Chinese Academy of Science, Shanghai, China; 4 Department of Nuclear Medicine, Shanghai Tenth People’s Hospital, Tongji University School of Medicine, Shanghai, China; 5 General Surgery, Ruijin Hospital & Institute of Pancreatic Diseases, Shanghai Jiaotong University School of Medicine, Shanghai, China; 6 Department of Oncology, Tongji Hospital, Huazhong University of Science and Technology, Wuhan, Hubei China

**Keywords:** Cancer microenvironment, Tumour immunology, Lung cancer

## Abstract

Immune checkpoint blockade (ICB) therapy targeting PD-1/PD-L1 has shown durable clinical benefits in lung cancer. However, many patients respond poorly to ICB treatment, underscoring an incomplete understanding of PD-L1 regulation and therapy resistance. Here, we find that MTSS1 is downregulated in lung adenocarcinoma, leading to PD-L1 upregulation, impairment of CD8^+^ lymphocyte function, and enhanced tumor progression. MTSS1 downregulation correlates with improved ICB efficacy in patients. Mechanistically, MTSS1 interacts with the E3 ligase AIP4 for PD-L1 monoubiquitination at Lysine 263, leading to PD-L1 endocytic sorting and lysosomal degradation. In addition, EGFR-KRAS signaling in lung adenocarcinoma suppresses MTSS1 and upregulates PD-L1. More importantly, combining AIP4-targeting via the clinical antidepressant drug clomipramine and ICB treatment improves therapy response and effectively suppresses the growth of ICB-resistant tumors in immunocompetent mice and humanized mice. Overall, our study discovers an MTSS1-AIP4 axis for PD-L1 monoubiquitination and reveals a potential combinatory therapy with antidepressants and ICB.

## Introduction

Lung cancer is the leading cause of cancer-related death globally^1^, and the patients often suffer from tumor relapse with current therapies, leading to poor 5-year survival rates^2^. Frequent activating mutations in signaling pathways such as Kirsten rat sarcoma (*KRAS*) and epidermal growth factor receptor (*EGFR*) are found in lung adenocarcinoma (LUAD), the most prevalent histological subtype of lung cancer^3^, and specific inhibitors of these oncogenic pathways have been developed for cancer treatment^3–5^. However, tumor resistance towards these inhibitors is often quickly acquired^6,7^. Recently, ICB therapy targeting the immune checkpoint PD-1/PD-L1 axis has provided a promising direction for lung cancer care^8,9^. ICB therapy demonstrates durable effects toward a subset of tumors, such as those with high nonsynonymous mutation burden^10^. However, the response rate in most patients is not satisfactory. Tumors with low PD-L1 expression are usually refractory to the therapy. In addition, ICB benefit is also limited in patients harboring *EGFR* mutations or anaplastic lymphoma kinase (*ALK*) rearrangements^11^. Therefore, there is an increasing demand for new therapeutic approaches to overcome ICB resistance. Understanding how the expression of checkpoint molecules is regulated would help uncover the underlying mechanisms of therapy responsiveness and bring new combinatory immune therapy.

The expression of PD-L1 is regulated at multiple levels. PD-L1 transcription and mRNA stability are regulated by prominent oncogenic pathways such as c-Myc, RAS, and EGFR^12–15^. In addition, PD-L1 protein undergoes various forms of post-translational modification, including phosphorylation^16,17^, glycosylation^17–19^, palmitoylation^20,21^, acetylation^22^, poly-ubiquitination^23^, and deubiquitination^24^, leading to subcellular translocation or changes of protein stability. PD-L1 is a member of type I transmembrane proteins, a family often found to be regulated by monoubiquitination-mediated endocytosis and subsequent recycling to the membrane or lysosomal degradation^25^. Recent studies have demonstrated the involvement of lysosome sorting in PD-L1 degradation^20,26^. In addition, PD-L1 is found to be subject to monoubiquitination^20,27^, which can be blocked by palmitoylation in its cytoplasmic domain^20^. However, the regulatory mechanisms and functional effects of PD-L1 monoubiquitination remain largely unknown.

MTSS I-BAR domain containing 1 (MTSS1), also known as missing in metastasis (MIM), is a multifunctional scaffold protein that is downregulated in multiple cancer types^28–33^ and suppresses tumor inhibition, progression, and metastasis^32–38^. MTSS1 interacts with a number of proteins involved in cytoskeleton organization, such as actin, cortactin, RhoA, and Rac^39–44^, to regulate cell motility and invasion. It can also suppress cell migration by enhancing ubiquitination of the chemokine receptor CXCR4 and its subsequent lysosomal degradation^45^. In addition, we found that MTSS1 physically interacts with the E3 ligase RBCK1 to induce ubiquitination of the NF-κB subunit p65, leading to inhibition of breast cancer cell stemness^38^. These recent studies indicate the versatile role of MTSS1 as a scaffold protein to regulate intrinsic malignant behaviors of cancer cells. Nevertheless, it is not known whether MTSS1 is involved in the regulation of tumor immunology. In this study, we show that MTSS1 modulates AIP4-mediated PD-L1 monoubiquitination and lysosomal degradation, resulting in changes in immune evasion and ICB responsiveness of LUAD.

## Results

### MTSS1 suppresses LUAD development in a host immunity-dependent manner

**Fig. 1:**
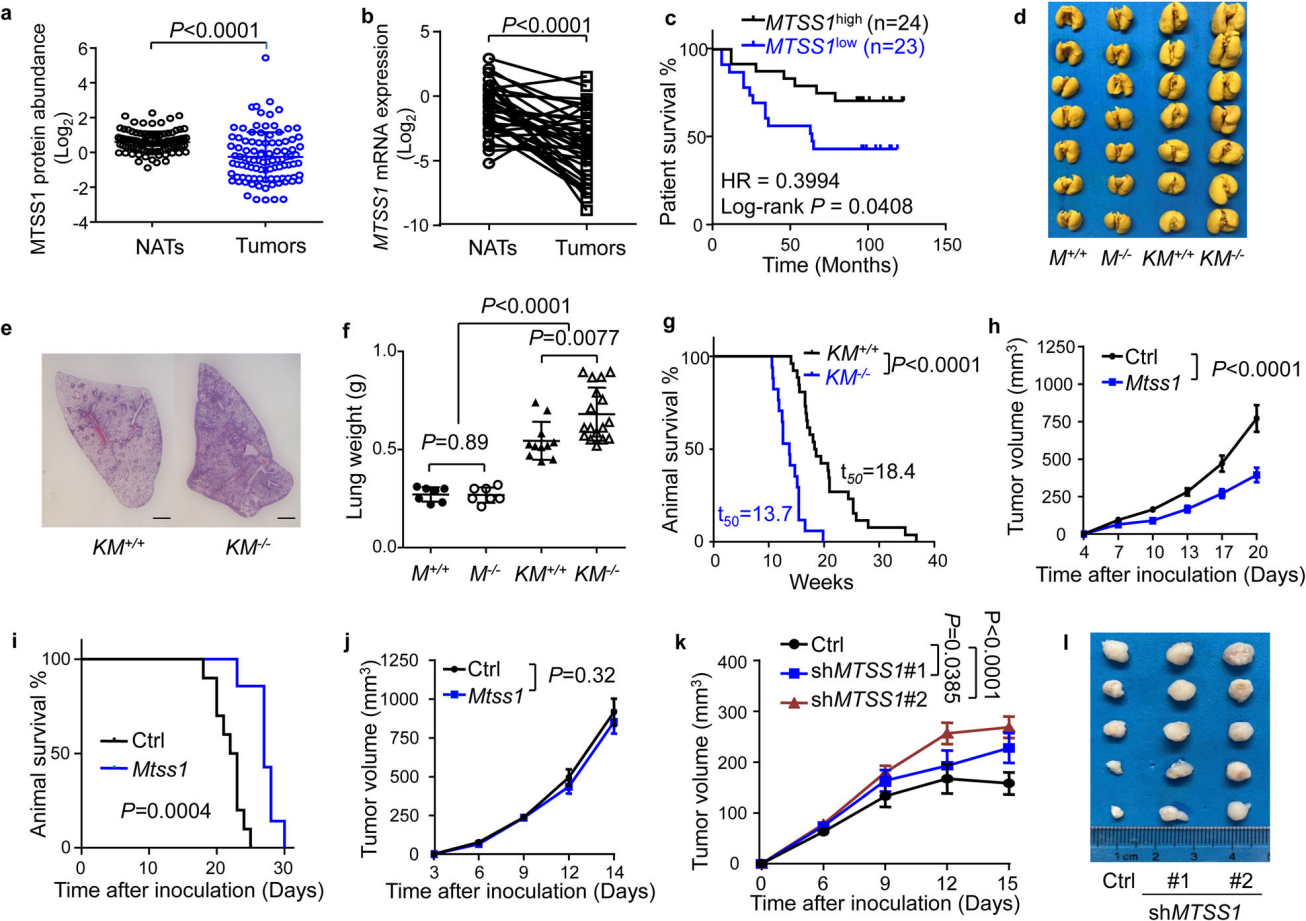
MTSS1 suppresses lung cancer development in immunocompetent hosts. **a** MTSS1 protein expression of human LUAD samples and paired normal adjacent tissues (NATs) in the CPTAC data portal^47^; *n* = 99 per group. **b** *MTSS1* mRNA levels of human LUAD samples and NATs in an STPH cohort (*n* = 36 per group). **c** Kaplan–Meier survival analysis of STPH patients based on *MTSS1* mRNA expression of the tumors. **d**–**g** Lung cancer development in mice of wild-type *Mtss1* (*M*^*+/+*^), *Mtss1* knockout (*M*^*–/–*^), *Kras*^*LSL-G12D*^ (*KM*^*+/+*^) or *Mtss1* knockout and *Kras*^*LSL-G12D*^ (*KM*^*–/–*^). *Kras*^*LSL-G12D*^ was activated by Cre adenoviral infection of the mice at the age of 6–8 weeks. Shown are images (**d**), hematoxylin and eosin (H&E) staining (**e**), and weights (**f**, *n* = 7, 7, 11, 17 respectively) of lungs harvested on week 16, and animal survival (**g**, *n* = 26 and 17 for *KM*^*+/+*^ and *KM*^*–/–*^). Scale bar (**e**), 1 mm. **h**, **i** Tumor growth of control and *Mtss1*-overexpressing LLC cells in immunocompetent C57BL/6J mice (**h**) and survival curves of the mice (**i**); *n* = 10 and 7 mice for the control and *Mtss1* groups. **j** Tumor growth of control and *Mtss1*-overexpressing LLC cells in nude mice (*n* = 7 per group). **k**, **l** Growth curves (**k**) and images (**l**) of H1975 tumors with or without *MTSS1* knockdown in PBMC-humanized NOG mice (*n* = 5 per group). Data are shown as means ± SEM (**h**, **j**, **k**) or means ± SD (**a**, **f**). *P* values were calculated by two-tailed paired (**a**, **b**) or unpaired (**f**) *t*-test, log-rank test (**c**, **g**, **i**), or two-way ANOVA (**h**, **j**, **k**).

Previous studies in our group have shown the critical roles of MTSS1 in the regulation of breast cancer progression^37,38^. Notably, a significant downregulation of MTSS1 in lung cancer was also observed. In analyses of several public clinical datasets of LUAD^46–48^, we found that *MTSS1* mRNA and protein levels were significantly lower in cancer tissues as compared with normal adjacent tissues (NATs), and *MTSS1* downregulation in LUAD was associated with poor patient survival (Fig. 1a and Supplementary Fig. S1a, b). We further analyzed *MTSS1* expression in a cohort of human LUAD samples with paired NATs and still observed obvious downregulation of *MTSS1* in LUAD (Fig. 1b). Concordantly, higher *MTSS1* expression in cancer tissues correlated to improved patient survival (Fig. 1c). These results indicate a suppressive role of MTSS1 in LUAD development.

Thus, we assessed the effect of *Mtss1* knockout in mice on lung cancer. The *Mtss1* knockout (*Mtss1*^*–/–*^) mice^38,49^ were crossed with the *Kras*^*LSL-G12D*^ mice, followed by intranasal instillation of Cre adenoviruses to activate inducible the *Kras*^G12D^ allele. *Mtss1* loss was confirmed in the *Kras*^*LSL-G12D*^; *Mtss1*^*–/–*^ (*KM*^*–/–*^) mice as compared to *Kras*^*LSL-G12D*^ mice (Supplementary Fig. S1c). Importantly, genetic ablation of *Mtss1* accelerated the development of *Kras*^G12D^-induced lung cancer in the mice, as evidenced by increased tumor burden in the lungs (Fig. 1d, e) and lung weights (Fig. 1f) in age-matched mice. The survival of the mice was also significantly shortened by *Mtss1* knockout (Fig. 1g).

We further ectopically expressed Mtss1 in Lewis lung carcinoma (LLC) cells (Supplementary Fig. S1d) and analyzed xenograft tumorigenesis by subcutaneous inoculation of the cells in mice. Mtss1 overexpression significantly inhibited tumor growth in immunocompetent C57BL/6J mice (Fig. 1h) and prolonged animal survival (Fig. 1i). Interestingly, we found that Mtss1 had no effect on tumor development in immunodeficient mice when the cells were inoculated into BALB/c nude mice (Fig. 1j), suggesting that the role of MTSS1 in LUAD is dependent on host immune system.

To confirm the effect of human immune cells on MTSS1, we used the humanized mouse model by engraftment of peripheral blood mononuclear cells (PBMC) in severely immunodeficient NOG mice (Supplementary Fig. S1e, f). *MTSS1* was knocked down in the human H1975 lung cancer cell line (Supplementary Fig. S1g), followed by inoculation of the cells into the PBMC-humanized mice or control NOG mice. In the mice with reconstitution of human immune cells, *MTSS1* knockdown obviously enhanced tumor growth (Fig. 1k, l). However, such an effect was not observed in the control immunodeficient NOG mice. Instead, a slight suppression of tumor growth was seen after the *MTSS1* knockdown (Supplementary Fig. S1h).

### *MTSS1* downregulation enhances PD-L1 protein expression and promotes immune evasion

**Fig. 2:**
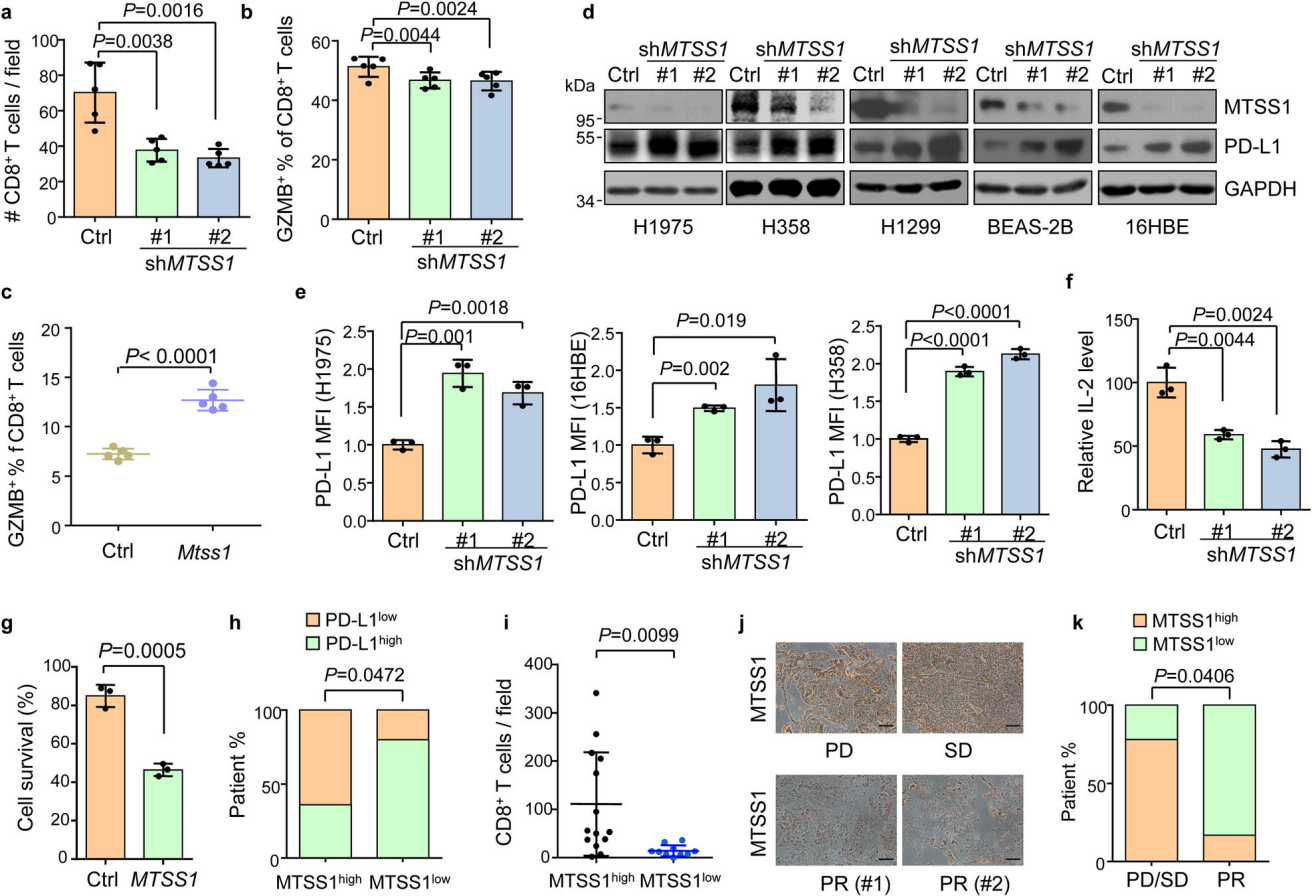
MTSS1 suppresses PD-L1 protein expression and immune evasion. **a**, **b** Quantification of immunohistochemistry staining of CD8^+^ T cells (**a**) and GZMB^+^CD8^+^ T cells (**b**) in H1975 tumors with or without *MTSS1* knockdown in PBMC-humanized NOG mice (*n* = 5 per group). **c** Flow cytometry analysis of GZMB^+^CD8^+^ T cells in control and *Mtss1*-overexpressing LLC tumor tissues (*n* = 5 per group). **d** MTSS1 and PD-L1 protein levels in the indicated normal lung bronchial and lung cancer cell lines after *MTSS1* knockdown. **e** Cell surface PD-L1 was quantified by flow cytometry mean fluorescence intensity (MFI) in lung cancer cell lines after *MTSS1* knockdown. **f** IL-2 levels in supernatants of Jurkat cells co-cultured with H1975 with or without *MTSS1* knockdown for 24 h. **g** Survival of H1975 cells with *MTSS1* overexpression after co-culturing with activated human T cells. **h**, **i** Quantification of immunohistochemistry staining of PD-L1 (**h**) and CD8^+^ T cells (**i**) in human LUAD samples with high (*n* = 14) and low (*n* = 10) MTSS1 expression. **j**, **k** Representative images (**j**) and quantitation (**k**) of MTSS1 immunostaining in LUAD patients displaying different responses to anti-PD1/PD-L1 therapy, including partial response (PR, *n* = 6), progressive disease (PD, *n* = 1) and stable disease (SD, *n* = 8). Scale bars, 100 μm. Data are shown as means ± SD (**a**–**c**, **e**–**g**, **i**). *P* values were calculated by a two-tailed *t*-test (**a**–**c**, **e**–**g**, **i**), or Fisher’s exact test (**h**, **k**).

The above data suggest a role of MTSS1 in the regulation of the tumor immune microenvironment. Notably, in a published clinical dataset^50^, *MTSS1* expression of human LUAD samples positively correlated with enrichment of the CD8^+^ T cell signature^51^ in the tumors (Supplementary Fig. S2a, b). Thus, we analyzed T cells in the H1975 tumor tissues with or without *MTSS1* knockdown grafted in PBMC-humanized mice by immunohistochemistry staining and flow cytometry. CD8^+^ T cell infiltration and activation were markedly suppressed in the tumors with *MTSS1* knockdown (Fig. 2a, b and Supplementary Fig. S2c). Conversely, an obvious upregulation of granzyme B (GZMB)^+^; CD8^+^ T cells were observed after Mtss1 overexpression (Fig. 2c and Supplementary Fig. S2d). MTSS1 downregulation resulted in the suppression of anti-tumor CD8^+^ T cells in LUAD.

In concordance with the suppressed CD8^+^ T cell activity, we found that *MTSS1* knockdown resulted in obvious upregulation of PD-L1 protein expression in H1975 cells (Fig. 2d), which was further confirmed in additional human non-small-cell lung cancer cell lines H358, H1299 and bronchial epithelium cell lines 16HBE, BEAS-2B (Fig. 2d). Flow cytometry analyses also demonstrated the increased levels of PD-L1 on the cell surface with *MTSS1* knockdown (Fig. 2e and Supplementary Fig. S2e). *Mtss1* knockout in mice also led to an upregulation of PD-L1 in lung tumors (Supplementary Fig. S1c). Reciprocally, MTSS1 overexpression reduced the protein levels of PD-L1 in lung cancer and bronchial epithelial cells (Supplementary Fig. S2f). In addition, when cancer cells were co-cultured with Jurkat T cells, *MTSS1* knockdown in H1975 significantly decreased IL-2 secretion from T cells (Fig. 2f). T cell-mediated killing of cancer cells was also suppressed by *MTSS1* knockdown and enhanced by MTSS1 overexpression (Fig. 2g and Supplementary Fig. S2g). In contrast, the expression of genes involved in the IFNγ signaling pathway or antigen presentation was not affected by MTSS1 (Supplementary Fig. S2h). Hence, MTSS1 inhibits PD-L1 expression and regulates tumor immune evasion.

We further investigated the relationship of MTSS1 expression with PD-L1 expression, lymphocyte infiltration, and response to ICB therapy in clinical LUAD samples. Consistent with the in vitro observations, immunohistochemistry staining revealed that LUAD samples with lower MTSS1 expression displayed significantly elevated PD-L1 levels and less infiltrated CD8^+^ T cells within the tumors (Fig. 2h, i and Supplementary Fig. S2i). More importantly, we analyzed the expression of MTSS1 in a cohort of LUAD patients who received ICB treatment and observed an indicative role of MTSS1 to therapy response. The patients with weaker MTSS1 expression displayed improved objective response rates to ICB therapy as compared to those with stronger MTSS1 expression (Fig. 2j, k), corroborating the role of MTSS1 to regulate PD-L1 expression and tumor immune evasion.

### MTSS1 interacts with PD-L1 and facilitates PD-L1 lysosomal degradation

**Fig. 3:**
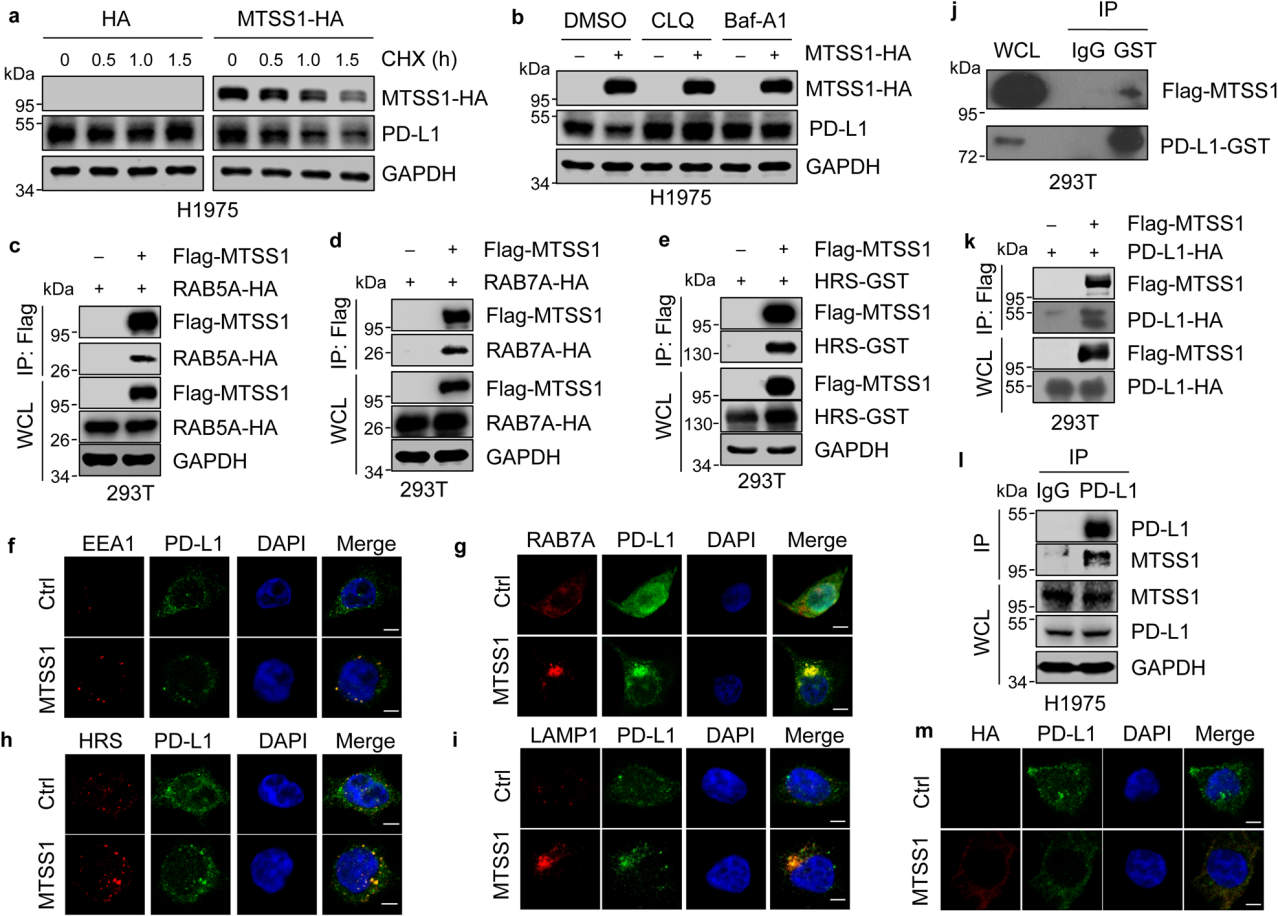
MTSS1 interacts with PD-L1 and promotes PD-L1 lysosomal degradation. **a**, **b** PD-L1 protein levels in H1975 cells with or without *MTSS1* overexpression with the treatment of cycloheximide (CHX, 100 μM) for the indicated duration (**a**), or lysosome inhibitors chloroquine (CLQ, 150 μM) or bafilomycin-A1 (Baf-A1, 0.4 μM) for 12 h (**b**). **c**–**e** Co-immunoprecipitation (Co-IP) of ectopically expressed MTSS1 with endocytic organelle markers RAB5A (**c**), RAB7A (**d**), and HRS (**e**) in 293 T. WCL, whole cell lysates. **f**–**i** Immunofluorescence (IF) analysis for colocalization of PD-L1 with endocytic organelle markers EEA1 (**f**), RAB7A (**g**), HRS (**h**), and LAMP1 (**i**) in H1975 cells with or without *MTSS1* overexpression. **j**, **k** Co-IP of ectopically expressed MTSS1 and PD-L1 in 293 T by detecting MTSS1 in PD-L1 (GST tagged) immunoprecipitates (**j**) and detecting PD-L1 in MTSS1 (Flag tagged) immunoprecipitates (**k**). **l** Co-IP of endogenous MTSS1 and PD-L1 in H1975. **m** IF analysis for MTSS1 and PD-L1 colocalization in H1975. Scale bars, 5 μm.

Next, we analyzed how MTSS1 regulates PD-L1. It was observed that the mRNA level of *PD-L1* was unaffected by MTSS1 in human and murine cancer cells (Supplementary Fig. S3a–d). Instead, MTSS1 impaired the stability of the PD-L1 protein when the cells were treated with cycloheximide (Fig. 3a and Supplementary Fig. S3e–j). Furthermore, treatment of cells with lysosome inhibitors chloroquine and bafilomycin-A1, but not with the proteasome inhibitor MG132 or the autophagy inhibitor Spautin-1^52^, restored the PD-L1 protein levels that were reduced by *MTSS1* overexpression (Fig. 3b and Supplementary Fig. S3k, l), suggesting the involvement of lysosome pathway in MTSS1-regulated PD-L1 degradation.

Membrane proteins can be internalized in an endocytic process, in which they are sorted sequentially into early endosomes, late endosomes, multivesicular bodies (MVBs), and then lysosomes for degradation. We found that MTSS1 physically interacted with the proteins involved in the formation of endocytic subcellular organelles, including RAB5A (early endosome), RAB7A (late endosome) and HRS (MVB) (Fig. 3c–e and Supplementary Fig. S3m). Immunofluorescent staining also confirmed the colocalization of MTSS1 with these markers in lung cancer cells (Supplementary Fig. S3n). More importantly, MTSS1 enhanced the presence of PD-L1 in endosomes, MVBs, and lysosomes, as evidenced by the colocalization of PD-L1 with the organelle markers EEA1, RAB7A, HRS, and LAMP1 (Fig. 3f–i). In addition, reciprocal co-immunoprecipitation assays demonstrated that ectopically expressed MTSS1 was able to bind to PD-L1 in 293 T cells (Fig. 3j, k). Physical interaction of endogenous MTSS1 to endogenous PD-L1 was also confirmed in H1975 cells (Fig. 3l). Concordantly, co-localization of MTSS1 with PD-L1 was observed in cancer cells (Fig. 3m). Taken together, these results showed that MTSS1 interacts with PD-L1 and promotes the endocytic trafficking of PD-L1, leading to its lysosomal degradation.

### MTSS1 promotes AIP4-mediated PD-L1 monoubiquitination at K263

**Fig. 4:**
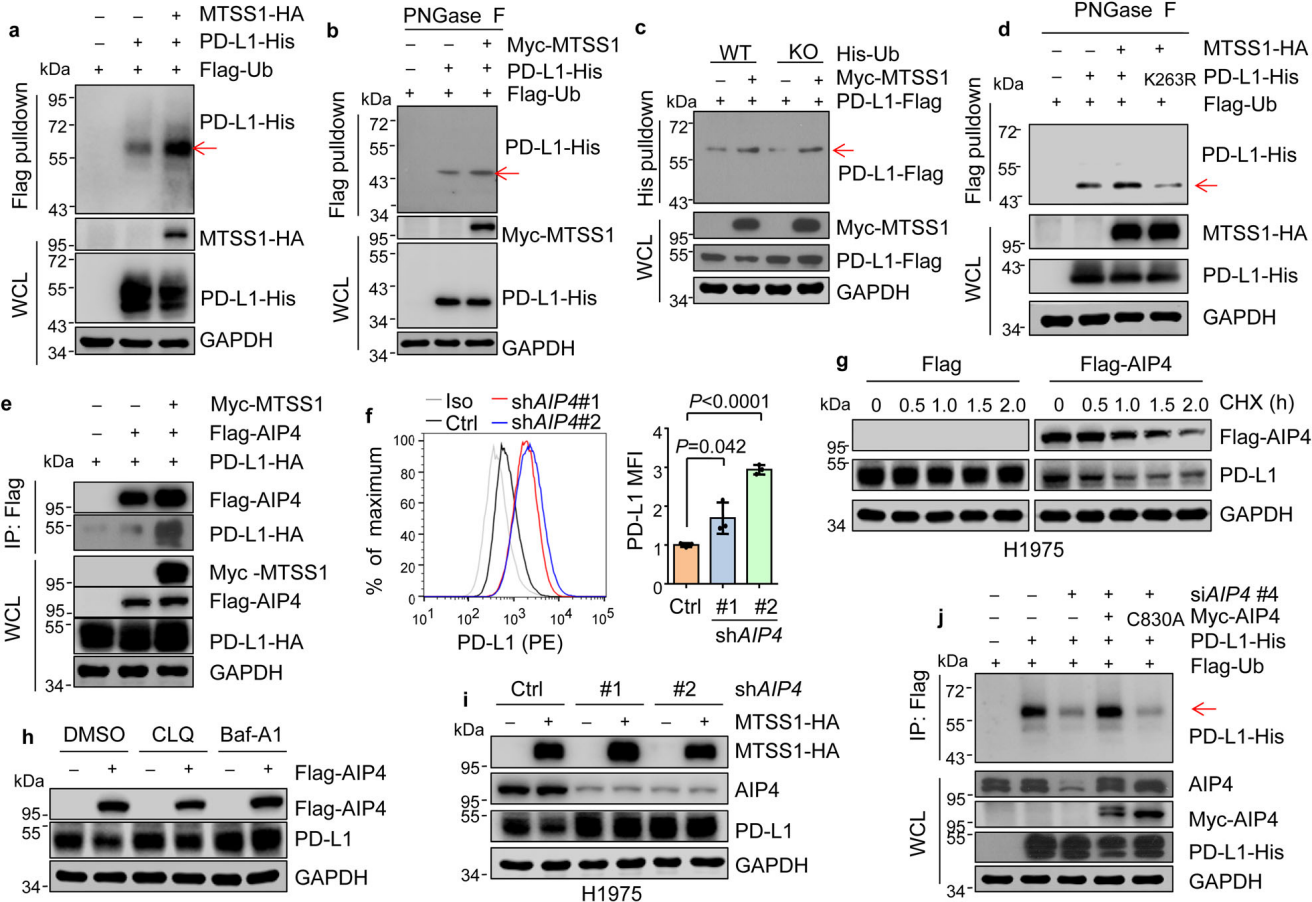
MTSS1 promotes AIP4-mediated PD-L1 monoubiquitination at K263. **a** PD-L1 ubiquitination analysis in HeLa cells with or without *MTSS1* overexpression. Note that PD-L1 in WCL appeared as multiple bands in 45–55 kDa, likely due to glycosylation. **b** PD-L1 ubiquitination analysis in HeLa cells with in vitro PNGase F treatment of the proteins before immunoblotting. **c** PD-L1 ubiquitination analysis in HeLa transfected with wild type (WT) or KO mutant ubiquitin (with all seven lysines mutated to arginines), and other indicated constructs. **d** Ubiquitination analyses of PD-L1 with Lysine 263 mutated to arginine (K263R) in 293 T with in vitro PNGase F treatment of the proteins before immunoblotting. **e** Co-IP of ectopically expressed AIP4 with PD-L1 in 293 T with or without *MTSS1* overexpression. **f** Flow cytometry analysis of cell surface PD-L1 in 16HBE with *AIP4* knockdown. Quantitation of PD-L1 mean fluorescence intensity (MFI) is shown on the right. Iso, isotype. **g**, **h** PD-L1 protein levels in H1975 cells after *AIP4* overexpression, with the treatment of cycloheximide (CHX, 100 μM) for the indicated duration (**g**), or chloroquine (CLQ, 150 μM) or bafilomycin-A1 (Baf-A1, 0.4 μM) for 12 h (**h**). **i** PD-L1 protein levels in H1975 with *AIP4* knockdown and/or *MTSS1* overexpression. **j** PD-L1 ubiquitination assay in Hela with knockdown of endogenous *AIP4* and overexpression of WT or C830A mutant *AIP4*. si*AIP4* (#4) targets the 3’UTR region of the endogenous *AIP4*. Data are shown as means ± SD, and *P* values were calculated by two-tailed unpaired *t*-test (**f**). Arrow points to monoubiquitinated PD-L1.

We further analyzed the mechanism of MTSS1 to induce PD-L1 lysosomal degradation. Previously it was reported that CMTM6 interacted with PD-L1 and reduced its lysosome degradation^26^. We found that MTSS1 had no effect on CMTM6 and PD-L1 interaction (Supplementary Fig. S4a). Furthermore, MTSS1 still inhibited PD-L1 expression when *CMTM6* was silenced (Supplementary Fig. S4b), indicating a CMTM6-independent mechanism of PD-L1 regulation by MTSS1. Membrane proteins are often labeled for endocytosis and lysosomal degradation by monoubiquitination^25^, and PD-L1 is found to be subject to monoubiquitination^20,27^. Thus, we analyzed the ubiquitination status of PD-L1. PD-L1 ubiquitination was enhanced by MTSS1 in HeLa cells. Importantly, we observed a distinct band of ubiquitinated PD-L1 protein with a molecular size slightly larger than that of un-ubiquitinated PD-L1 (Fig. 4a), indicating PD-L1 monoubiquitination. The same phenomenon was also observed for endogenous PD-L1 protein in H1975 cells (Supplementary Fig. S4c). PD-L1 is known to be heavily glycosylated^17^. In vitro treatment with the recombinant glycosidase, PNGase F further collapsed the ubiquitinated PD-L1 species into a single band at approximately 45 kDa (Fig. 4b), consistent with the reported size of monoubiquitinated PD-L1^27^. In addition, MTSS1-enhanced ubiquitination of PD-L1 was still observed when all the seven lysines of ubiquitin were mutated (Fig. 4c), further confirming the monoubiquitination, instead of polyubiquitination, of PD-L1.

We further analyzed the modification site of PD-L1 monoubiquitination. Deletion of the cytoplasmic carboxyl fragment of PD-L1 completely abolished PD-L1 monoubiquitination (Supplementary Fig. S4d), suggesting that the monoubiquitination site resides in the carboxyl tail of PD-L1. Next, mass spectrometry (MS) analysis of the ubiquitinated PD-L1 pulldown identified a monoubiquitination site at Lysine 263 in the carboxyl fragment (Supplementary Fig. S4e, f). Concordantly, mutating Lysine 263 to arginine (K263R) of PD-L1 largely abolished the MTSS1-regulated monoubiquitination of PD-L1 (Fig. 4d). Thus, these data demonstrated that MTSS1 promotes PD-L1 monoubiquitination at Lysine 263.

MTSS1 is a scaffold protein without ubiquitin ligase activity^53^. Thus, we aimed to identify the E3 ligase for PD-L1 in order to investigate how MTSS1 regulates PD-L1 ubiquitination. By screening several E3 ligases that have been reported to regulate membrane protein monoubiquitination^54^, we found that the HECT ubiquitin ligase AIP4 (also named ITCH) interacted with PD-L1 (Supplementary Fig. S5a). The physical binding of AIP4 to PD-L1 was further confirmed in multiple cell lines by reciprocal co-immunoprecipitation with both ectopically expressed and endogenous proteins (Supplementary Fig. S5b, c). Interaction between MTSS1 and AIP4 was also observed in cancer cells (Supplementary Fig. S5d, e). Analysis of a published single-cell RNA-seq (scRNA-seq) dataset of murine LUAD^55^ revealed the expression of both *Mtss1* and *Aip4* in tumor cells (Supplementary Fig. S5f–h). Mutual colocalization of AIP4, MTSS1, and PD-L1 in cells was confirmed (Supplementary Fig. S5i). Sequential immunoprecipitation assay confirmed the presence of the three proteins in one complex (Supplementary Fig. S5j). Importantly, MTSS1 prominently enhanced the binding of AIP4 to PD-L1 (Fig. 4e and Supplementary Fig. S5k). In addition, *AIP4* knockdown dramatically increased the expression level of PD-L1 in the cells (Supplementary Fig. S6a) and on the cell surface (Fig. 4f). In contrast, *AIP4* overexpression diminished the protein level of PD-L1 in human and murine LUAD cell lines (Supplementary Fig. S6b). AIP4 regulated PD-L1 by promoting its protein degradation (Fig. 4g and Supplementary Fig. S6c), but not by regulating the mRNA level (Supplementary Fig. S6d). Treatment of cells with the lysosome inhibitors chloroquine and bafilomycin-A1, but not with the proteasome inhibitor MG132, rescued PD-L1 expression that was inhibited by *AIP4* overexpression (Fig. 4h and Supplementary Fig. S6e).

In concordance with the role of AIP4 to mediate MTSS1-regulated PD-L1 monoubiquitination, *AIP4* knockdown dramatically reduced PD-L1 monoubiquitination (Supplementary Fig. S6f, g) and enhanced PD-L1 stability in H1975 (Fig. 4i and Supplementary Fig. S6h, i). Notably, MTSS1 was not able to induce PD-L1 monoubiquitination when *AIP4* was knocked down (Supplementary Fig. S6g). Exogenous expression of wild-type *AIP4*, but not the catalytically inactive C830A mutant^56^, in *AIP4*-knockdown cells restored the monoubiquitination of PD-L1 (Fig. 4j). Collectively, these data suggested that MTSS1 regulates PD-L1 monoubiquitination by promoting the interaction of PD-L1 and the E3 ligase AIP4.

### EGFR-KRAS signaling stabilizes PD-L1 stability by suppressing MTSS1

**Fig. 5:**
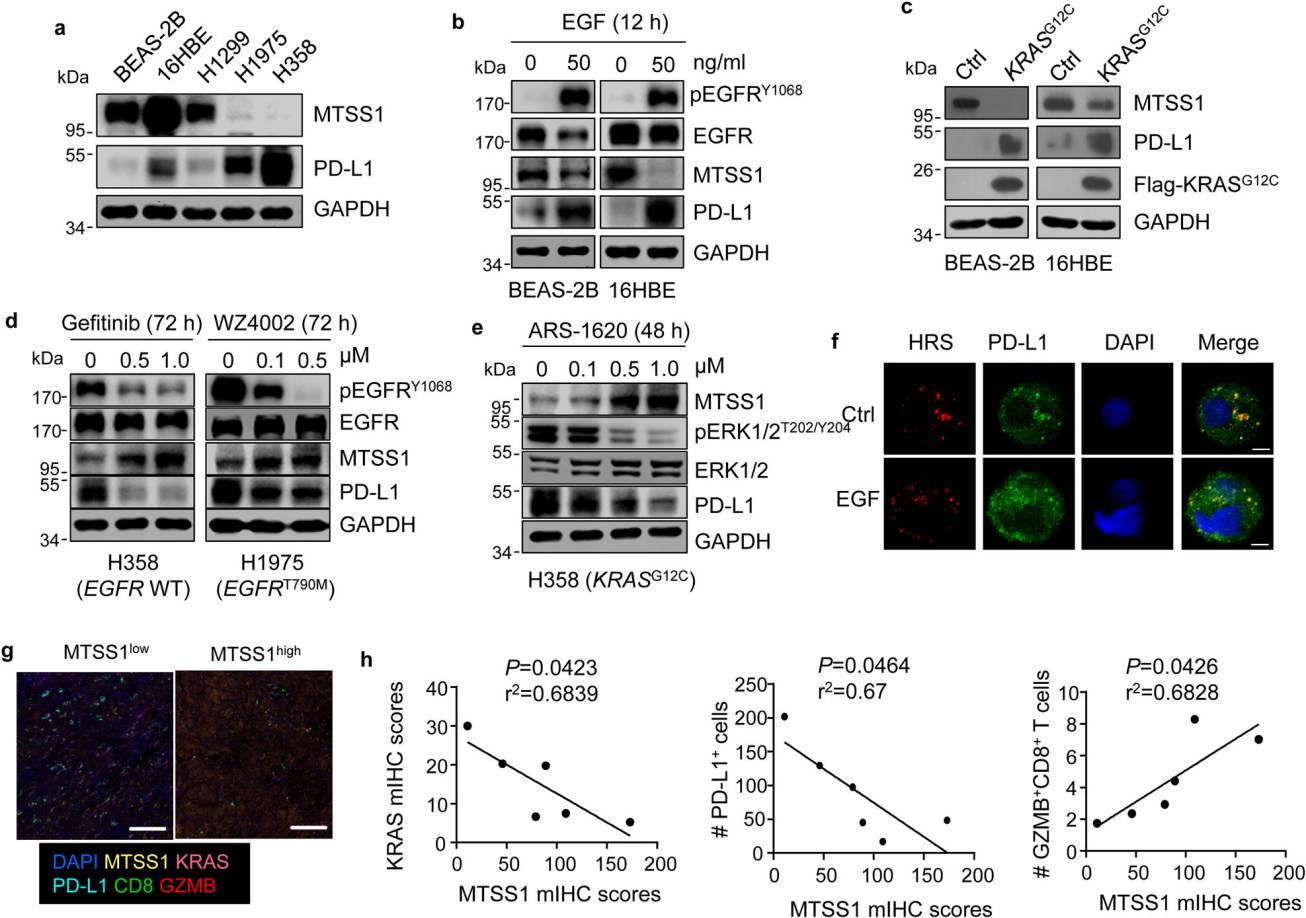
EGFR–KRAS signaling suppresses MTSS1 expression and PD-L1 monoubiquitination. **a** MTSS1 and PD-L1 expression in indicated normal bronchial epithelial cells (16HBE and BEAS-2B) and lung cancer cells (H1299, H1975 harboring *EGFR*^T790M^ and H358 harboring *KRAS*^G12C^). **b**, **c** Immunoblots of BEAS-2B and 16HBE cells treated with EGF for 12 h (**b**), or after *KRAS*^G12C^ overexpression (**c**). **d** Immunoblots of H358 treated with Gefitinib for 72 h or H1975 cells treated with WZ4002 for 72 h. **e** Immunoblots of H358 treated with various doses of ARS-1620 for 48 h. **f** IF analyses for colocalization of PD-L1 with HRS in 16HBE with or without 50 ng/mL EGF treatment for 15 min. Scale bars, 5 μm. **g**, **h** Representative mIHC staining images (**g**) and correlation of MTSS1 staining scores with KRAS scores, PD-L1^+^ cells, and GZMB^+^CD8^+^ T cells (**h**) in human LUAD samples (*n* = 6). Scale bars, 100 μm. *P* values were calculated by Pearson correlation analysis (**h**).

*KRAS* and *EGFR* are among the most frequent mutated genes with critical oncogenic roles in LUAD^3^. Interestingly, we observed that MTSS1 protein expression was substantially lower in human LUAD cell lines with *EGFR* and *KRAS* mutation, including H1975 (*EGFR*^T790M^) and H358 (*KRAS*^G12C^) (Fig. 5a), implying that MTSS1 might be modulated by the EGFR–KRAS signaling. To test this hypothesis, we treated normal human lung bronchial epithelial cell lines (16HBE, BEAS-2B) with EGF and found that MTSS1 protein expression was significantly downregulated by the activation of EGFR signaling (Fig. 5b). *KRAS*^*G12C*^ overexpression in these cells also inhibited MTSS1 (Fig. 5c). Along this line, treating H358 cells with the EGFR inhibitor Gefitinib resulted in MTSS1 upregulation (Fig. 5d). However, in H1975 cells harboring the Gefitinib-resistant *EGFR*^T790M^ mutation, only the mutation-selective, second-generation EGFR inhibitor WZ4002^57^, but not Gefitinib, was able to elevate MTSS1 (Fig. 5d and Supplementary Fig. S7a). Furthermore, the *KRAS*^G12C^-selective inhibitor ARS-1620^4^ regulated MTSS1 in *KRAS*^G12C^-harboring H358 cells, but not in *KRAS*^G12S^-harboring A549 cells (Fig. 5e and Supplementary Fig. S7b). However, the ERK1/2 inhibitor SCH772984 or the PI3K inhibitor BKM120 did not obviously alter the expression of MTSS1 (Supplementary Fig. S7c, d), indicating that the EGFR–KRAS signaling regulates MTSS1 protein via downstream pathways other than RAF–MAPK–ERK or PI3K–AKT. Additionally, EGFR–KRAS signaling regulated MTSS1 by promoting its protein degradation, but not at the transcription level (Supplementary Fig. S7e–g). The detailed mechanism of EGFR–KRAS to regulate MTSS1 is to be further studied.

Together with MTSS1 suppression, EGFR–KRAS also enhances the protein level of PD-L1 (Fig. 5a–e). In addition, we observed that *KRAS*^*G12C*^ overexpression and EGF treatment attenuated the interaction of MTSS1 with PD-L1 (Supplementary Fig. S7h), leading to suppression of PD-L1 monoubiquitination (Supplementary Fig. S7i) and MVB sorting (Fig. 5f). Importantly, *MTSS1* overexpression partially reduced PD-L1 expression that was enhanced by EGF (Supplementary Fig. S7j). Combining ERK/PI3K inhibition and MTSS1 overexpression was able to completely block the effect of EGFR activation on PD-L1 upregulation (Supplementary Fig. S7j). Finally, a multiplex immunohistochemistry staining (mIHC) analysis of human LUAD samples revealed lower KRAS and PD-L1 expression in MTSS1-expressing tumors, together with the enhanced presence of GZMB^+^CD8^+^ T cells (Fig. 5g, h). Thus EGFR–KRAS signaling stabilizes PD-L1 protein, at least partially, by suppressing MTSS1 protein expression in lung cancer.

### Combinatory clomipramine and ICB treatment effectively suppresses LUAD

**Fig. 6:**
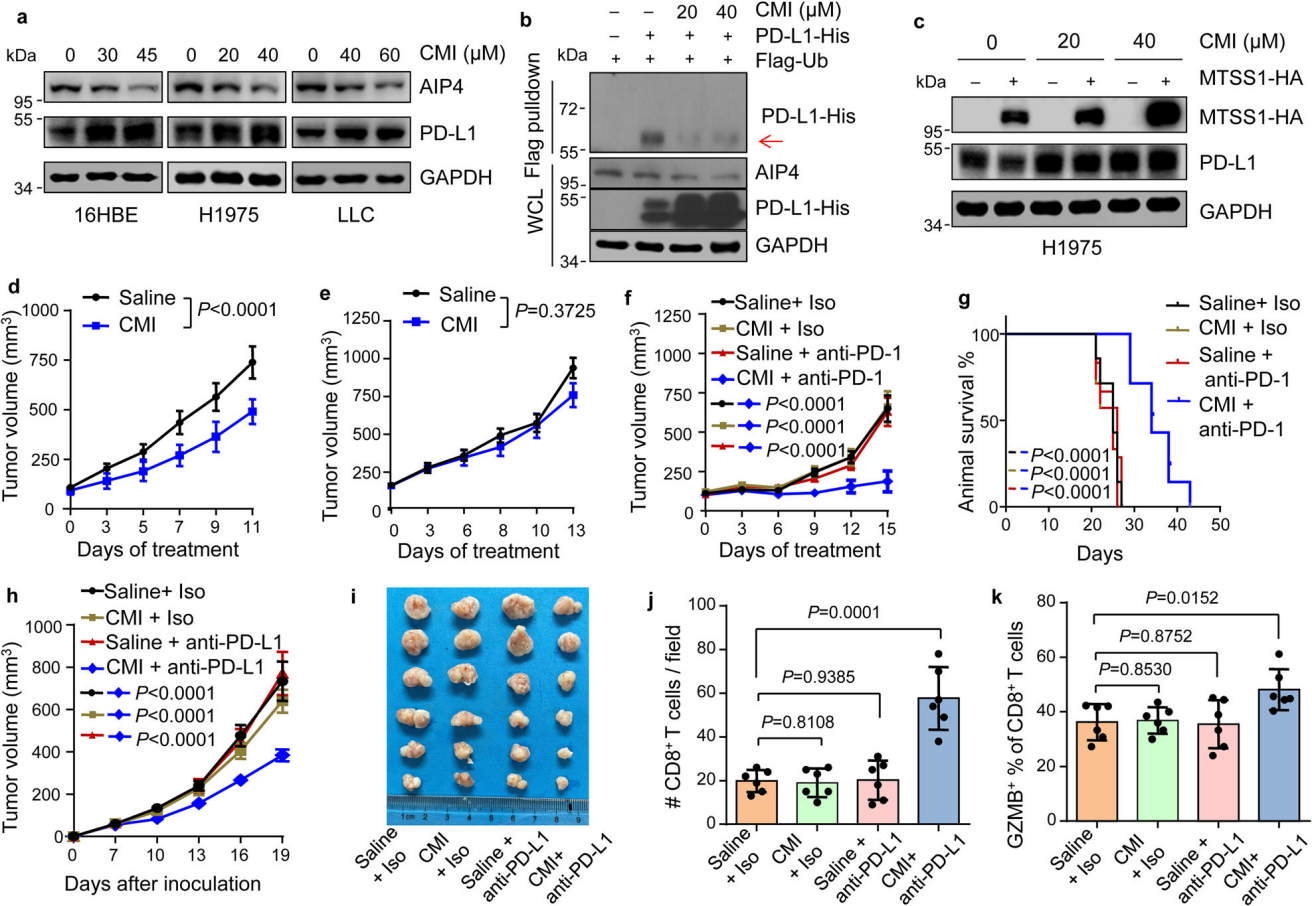
Combinatory clomipramine and ICB treatment effectively suppress lung cancer. **a** Immunoblots of PD-L1 and AIP4 in the indicated cell lines treated with clomipramine (CMI) in various concentrations. **b** PD-L1 ubiquitination in 293 T after CMI treatment. Arrow points to monoubiquitinated PD-L1. **c** Immunoblots of H1975 with *MTSS1* overexpression and/or CMI treatment. **d**, **e** Growth of LLC xenograft tumors in nude mice (**d**, *n* = 8 and 9 for saline and CMI groups, respectively) and immunocompetent C57BL/6J mice (**e**, *n* = 7 per group) with the treatment of CMI. Saline was used as treatment control. **f**, **g** Growth of LLC xenograft tumors in C57BL/6 J mice (**f**, *n* = 7, 7, 6 and 7 respectively) and animal survival (**g**) with the treatment of CMI and/or anti-PD-1 (RMP1-14). Saline and antibody isotype (Iso) were used as treatment control. **h**–**k** Treatment of PMBC-humanized NSG mice bearing H1975 xenograft tumors with CMI and/or anti-PD-L1 (Atezolizumab). Shown are tumor growth (**h**), tumor images (**i**), quantification of immunohistochemistry staining of CD8^+^ T cells (**j**) and GZMB^+^ CD8^+^ T cells (**k**) in the tumors (*n* = 6 mice per group). Data are shown as means ± SEM (**d**–**f**, **h**) or means ± SD (**j**, **k**). *P* values were calculated by a two-tailed *t*-test (**j**, **k**), log-rank test (**g**), or two-way ANOVA (**d**–**f**, **h**).

The critical role of the MTSS1–AIP4 axis on PD-L1 stability suggested that targeting MTSS1-AIP4 might have an effect on PD-L1 expression and ICB response. Clomipramine is an FDA-approved tricyclic antidepressant (TCA) drug and was also found to exert a tumor-suppressing effect by inhibiting autophagic flux^58,59^ and cancer cell stemness^60^. Therefore, clomipramine and similar TCA drugs are under clinical investigation for cancer treatment^58,61^. Indeed, we also found an obvious effect of clomipramine to inhibit LUAD cell growth and tumorsphere formation (Supplementary Fig. S8a, b). Interestingly, clomipramine is also an AIP4 inhibitor by blocking AIP4 ubiquitin transthiolation in an irreversible manner^62^. Concordant to the role of AIP4 on PD-L1 monoubiquitination, clomipramine treatment of lung epithelial and cancer cells effectively inhibited PD-L1 monoubiquitination and enhanced PD-L1 protein abundance in the cells and on the cell surface (Fig. 6a, b and Supplementary Fig. S8c, d). Notably, MTSS1 was no longer able to inhibit PD-L1 with clomipramine treatment (Fig. 6c). Further, we tested the in vivo anti-tumor efficacy of clomipramine (Supplementary Fig. S8e). Clomipramine treatment displayed no obvious side effect on animal body weight (Supplementary Fig. S8f), consistent to the observation of the drug treatment of human patients^61^. More importantly, in vivo clomipramine treatment of immunodeficient nude mice with LLC tumors resulted in obvious tumor retardation (Fig. 6d), but had no effect on tumor growth in immunocompetent mice (Fig. 6e), suggesting that immune regulation compromises the anti-tumor efficacy of clomipramine.

The above observation also indicates a potential combinatory approach with clomipramine and ICB therapy for cancer treatment. Then we tested this strategy in immunocompetent mice with LLC tumors (Supplementary Fig. S8g, h). Again, single clomipramine treatment was not effective. In addition, LLC tumors were not responsive to ICB treatment alone with the anti-PD-1 antibody RMP1–14, concordant to a previous report^63^. Notably, a combination of clomipramine and anti-PD-1 treatment substantially improved the therapy efficacy, with marked tumor suppression (Fig. 6f) and prolonged animal survival (Fig. 6g).

LUAD with *EGFR* mutations is known to respond poorly to ICB, possibly due to the lack of concurrent PD-L1 expression and high levels of CD8^+^ lymphocyte infiltration in tumors^11^. Thus, we tested whether the combinatory strategy could improve ICB efficacy toward human lung tumors with EGFR mutations in humanized mice. Xenograft tumors were established with human *EGFR*^T790M^-harboring H1975 lung cancer cells in PBMC-humanized NSG mice, followed by single or combinatory treatment of clomipramine and the anti-PD-L1 drug Atezolizumab (Supplementary Fig. S8i). The treatments displayed no obvious side effects (Supplementary Fig. S8j). Importantly, H1975 tumors barely responded to either the antidepressant or Atezolizumab, but were substantially suppressed by the dual treatment (Fig. 6h, i). The dual treatment significantly enhanced the infiltration and activation of CD8^+^ T cells in the tumors (Fig. 6j, k and Supplementary Fig. S8k), leading to obvious tumor retardation (Fig. 6h, i). Thus, a combination of clomipramine and ICB may be a promising approach to ameliorate ICB resistance in lung cancer.

## Discussion

Endocytic sorting for recycling to the membrane or lysosome degradation represents a critical regulatory process for cell surface proteins, especially receptors, and monoubiquitination often triggers their endocytosis^25,54^. PD-L1 has been intensively studied for its multiple forms of post-translational modifications^16–24^; however, its monoubiquitination is under-investigated. Previous studies have shown that PD-L1 is monoubiquitinated, and its ubiquitination can be blocked by the protein-stabilizing palmitoylation^20,27^, but the monoubiquitination process remained largely elusive. In addition, the effects of monoubiquitination on PD-L1 fate and tumor immune evasion were unknown. Here we show that MTSS1 facilitates the interaction of PD-L1 with the E3 ligase AIP4 and promotes PD-L1 monoubiquitination at Lysine 263, leading to internalization, endosome trafficking, and lysosomal degradation of PD-L1. MTSS1 downregulation in tumor cells resulting from EGFR–KRAS activation stabilizes PD-L1 and enhances immune evasion (Supplementary Fig. S8l). Our study will enrich the understanding of the regulation of PD-L1 intracellular dynamics and immune evasion of cancer. Additionally, our study demonstrates the correlation of MTSS1 expression with ICB efficacy and discovers a combinatory approach to improve ICB response, and thus may have important implications on cancer therapy.

Our analysis identifies the monoubiquitination of PD-L1 on Lysine 263. Notably, Lysine 263 of PD-L1 is also known to be subject to p300-mediated acetylation, which affects the nuclear translocation of PD-L1 and the immune response of cancer cells^22^. Lysine acetylation often competes with ubiquitination to regulate the stability or subcellular localization of non-histone proteins^64^. The mutual regulation of various modifications on this critical residue, and the influence on PD-L1 abundance and functionality, are worthy of further investigation.

The regulation of MTSS1 and PD-L1 monoubiquitination by EGFR–KRAS signaling underscores the clinical relevance of MTSS1 in LUAD. Notably, *MTSS1* overexpression only partially suppressed PD-L1 induction by EGF, indicating additional mechanisms of PD-L1 regulation by EGFR signaling. Indeed, previous studies have shown that EGFR–KRAS regulates PD-L1 transcription and mRNA stability^15,57^. EGFR–KRAS signaling may also promote PD-L1 protein expression through the downstream PI3K-ATK pathway^65^. These observations demonstrate the multifaceted regulation of PD-L1 expression by EGFR–KRAS signaling. In addition, we observed that EGF treatment reduced the protein stability, but not the mRNA transcription of *MTSS1* (Supplementary Fig. S7e–g), indicating a post-translational regulation. However, inhibiting the two main EGFR–RAS downstream pathways, RAF–MAPK–ERK and PI3K–AKT, had no effects on MTSS1 expression, suggesting other downstream effectors to mediate MTSS1 regulation. Nevertheless, the detailed mechanism for MTSS1 regulation by EGFR–KRAS is yet to be further investigated.

The improved efficacy of combinatory treatment with clomipramine and ICB is of particular clinical relevance. First, clomipramine and other TCA drugs are being clinically investigated for cancer treatment^58,61^, owing to their anti-tumor potentials to suppress cancer cell survival and stemness^58–60^ and the observed negative correlation of drug use with cancer incidence in human^66^. However, the clinical efficacy of clomipramine is currently not conclusive, with only anecdotal evidence. Our data showing the varied effects of clomipramine in different cancer models indicate the dual role of clomipramine on cancer cells and the immune microenvironment, and thus ICB therapy may provide an option to circumvent the side effect of clomipramine on immune evasion. Secondly, ICB fails to achieve durable benefits in a subset of LUAD. The patient with EGFR mutations is resistant to ICB therapy, possibly due to the lack of concurrent PD-L1 expression and lymphocyte infiltration in tumors^11^. Supplementing ICB with clomipramine treatment could enhance CD8^+^ T cell infiltration and activation for *EGFR*^T790M^-harboring tumors and effectively suppress several ICB-resistant tumor models. Thus, the antidepressant and ICB can complement each other in a combinatory approach for some treatment-resilient lung cancers. In addition, the combinatory approach could have another benefit, as depression is common in cancer patients. It is reported that more than 44% of lung cancer patients may suffer from depression, and depression symptoms are associated with increased mortality^67,68^. The treatment strategy may also help ameliorate the quality of life that is affected by depression in addition to suppressing tumors.

## Materials and methods

### Cell cultures, transfection, and virus infection

BEAS-2B, H1975, H358, H1299, A549, LLC, Jurkat, HeLa, and 293 T cell lines were purchased from the Cell Bank of Type Culture Collection of the Chinese Academy of Sciences. Cells were cultured in RPMI-1640 (H1975, H358, H1299, Jurkat, and 16HBE), DMEM (LLC, HeLa, and 293 T), F-12K (A549) or Bronchial Epithelial Cell Medium (BEAS-2B) supplemented with 10% fetal bovine serum (FBS), penicillin (100 units/mL) and streptomycin (100 μg/mL). All cell lines were confirmed as mycoplasma free by mycoplasma PCR tests. Plasmid transfection was performed using Lipofectamine 2000 (Invitrogen) in Opti-MEM (Gibco) media according to the manufacturer’s instructions.

### Reagents, plasmids, and antibodies used in this study

The reagents used in this study and their sources are listed in Supplementary Table S1. The pLVX-Flag, pLVX-HA, pLVX-Myc, and pLVX-GST vectors were used for the expression of human *MTSS1*, *AIP4*, *PD-L1*, *Ubiquitin*, *KRAS*^G12C^, *CBL*, *NEDD4*, *NEDD4L* and *SH3RF1*, *PD-L1-ΔC*, *RAB5A*, *RAB7A*, *RAB7B*, *AIP4-C830A*, *HRS*, *PD-L1*^K263R^. The pLVX- EF1a-Flag vector was used for the expression of murine *Mtss1* and *Aip4*. The pLKO.1-puromycin and pLKO.1-blasticidin (Addgene) vectors were used for the knockdown of *MTSS1* and *AIP4*, respectively. The sequences of siRNAs and shRNAs used in this study are provided in Supplementary Table S2. The pIP-His-Ubiquitin-WT and pIP-His-Ubiquitin-KO (all K to R mutants) plasmids were a gift from the laboratory of Bing Li at Shanghai Jiaotong University. The antibodies used in this study and their sources are listed in Supplementary Table S3. Information about the primers used in this study is provided in Supplementary Table S4.

### Immunoprecipitation

Cells were collected and then lysed with IP buffer (150 mM NaCl, 20 mM HEPES pH 7.4, 1% Triton X-100, 12.5 mM β-glycerophosphate, 1.5 mM MgCl_2_, 2 mM EGTA) with inhibitors (10 mM NaF, 1 mM PMSF, 1 mM Na_3_VO_4_ and Protease inhibitor cocktail). Equal amounts of protein were incubated with the primary antibody or control antibody for overnight at 4 °C, followed by incubation with protein A or protein G dynabeads (GE Life Sciences) for 2 h at 4 °C. The samples were washed three times with IP buffer before being resolved by sodium dodecyl sulphate-polyacrylamide gel electrophoresis (SDS-PAGE) and immunoblotted. For Flag magnetic bead immunoprecipitation, cells were lysed, and equal amounts of protein were incubated with Flag magnetic beads (M8823, Sigma) for 2 h at room temperature, followed by incubation with 5 packed gel volumes of 3× Flag elution solution (150 ng/mL final concentration) for 45 min at 4 °C. The supernatants were boiled for 10 min at 95 °C before being resolved by SDS-PAGE and immunoblotted.

### In vivo monoubiquitination assays

The Signal-Seeker Ubiquitin Enrichment Kit (BK161, Cytoskeleton) was used according to the manufacturer’s instructions to pull down ubiquitinated proteins. Briefly, H1975 cells with or without *MTSS1* overexpression were collected and lysed with BlastR™ lysis buffer with inhibitors (de-ubiquitinase inhibitor, Cat # NEM09BB; protease inhibitor cocktail, Cat # PIC02). The lysates were transferred into BlastR™ filters and diluted with BlastR™ dilution buffer to the final volume. Equal amounts of protein were incubated with 20 µL ubiquitination affinity beads or control beads for 2 h at 4 °C. The beads were washed 3 times with BlastR-2™ wash buffer, followed by incubation with 30 µL elution buffer for 5 min at room temperature. The precipitates were collected by the spin columns provided in the kit and were boiled for 10 min at 95 °C before being resolved by SDS-PAGE and immunoblotted.

For His-tagged ubiquitin pulldown with Ni–NTA beads, HeLa cells were transfected with wild-type (His–Ubiquitin–WT) or lysine-mutated (His–Ubiquitin–KO) ubiquitin and the other plasmids for 60–72 h. Cells were harvested and resuspended in Buffer A (6 M guanidine–HCl, 0.1 M Na_2_HPO_4_/NaH_2_PO_4_, 10 mM imidazole pH 8.0) with inhibitors (10 mM NaF, 1 mM PMSF, 1 mM Na_3_VO_4_, 5 mM N-Ethylmaleimide and Protease inhibitor cocktail). The lysates were sonicated (75 W, 2 s, 5 s, 3 min) before mixing with Ni–NTA beads (QIAGEN) by rotating at room temperature for 3 h. Subsequently, the His pull-down products were washed twice with buffer A, twice with buffer A/TI (1 volume buffer A and 3 volumes buffer TI), and once with buffer TI (25 mM Tris-HCl and 20 mM imidazole, pH 6.8). Then elution buffer (0.2 M imidazole, 5% w/v SDS, 0.15 M Tris-Cl, pH 6.8) was added and incubated for 20 min at room temperature. The supernatants were boiled for 10 min at 95 °C before being resolved by SDS-PAGE and immunoblotted.

For Flag-tagged ubiquitin pulldown, 293 T or HeLa cells transfected with Flag-ubiquitin were lysed with IP buffer with inhibitors (10 mM NaF, 1 mM PMSF, 1 mM Na_3_VO_4_, 5 mM N-Ethylmaleimide and Protease inhibitor cocktail). The subsequent immunoprecipitation was performed as described above. For PD-L1 deglycosylation, immunoprecipitates or whole cell lysates were treated using PNGase F according to the manufacturer’s instructions. Finally, samples were boiled for 10 min at 95 °C before being resolved by SDS-PAGE and immunoblotted.

### Flow cytometry

For cell culture analysis, cells with *MTSS1* or *AIP4* knockdown were cultured, suspended, and stained with anti-human CD274-PE or control antibody for 45 min at 4 °C. After washing three times with PBS, cells were analyzed on a Gallios analyzer (Beckman Coulter Life Sciences). For tumor analysis, tumors were excised from euthanized mice, cut into pieces, and then digested with buffer (RPMI 1640 containing 2.5 mg/mL Dispase II, 2.5 mg/mL collagenase IV, and 50 µg/mL DNAse I) at 37 °C for 30–60 min. The cell suspension was filtered with a 70-µm strainer before erythrocyte lysis. The cells were cultured in RPMI-1640 with 10% FBS and Cell Stimulation Cocktail (plus protein transport inhibitors) at 37 °C for 4 h. The samples were incubated with a buffer mix (PBS containing 10% FBS, 1 µL CD16/CD32 Fc blocking antibody, and 0.1 µL Fixable Viability Dye eFluor™ 780) at 4 °C for 15 min and then incubated with CD45, B220, CD11B, CD3, and CD8 antibodies at 4 °C for 30–45 min. Granzyme B was detected by intracellular staining. Finally, the cells were analyzed on CytoFLEX LX analyzer (Beckman Coulter Life Sciences). The gating strategy is shown in Supplementary Fig. S2d. Data were analyzed with FlowJo v.10 (FlowJo LLC). The antibodies used in this study and their sources are listed in Supplementary Table S3.

### Immunofluorescence staining

Cells were cultured on cover slides in 24-well plates for 24–48 h, fixed with 4% paraformaldehyde for 10 min, permeabilized with 0.1% Triton X-100 for 10 min, and blocked with 3% bovine serum albumin (BSA) for 1 h at room temperature. Then cells were incubated with the primary antibody at 4 °C overnight and washed three times with PBS before being incubated with the fluorescent conjugated secondary antibody for 1 h at room temperature. Finally, cells were stained with DAIP for 10 min, mounted (S3023, Dako) at room temperature, and imaged on confocal microscopy (Zeiss LSM880). All data were analyzed with the ZEN (blue edition) 2.6 software (ZEISS).

### Clinical samples and immunohistochemistry

The LUAD specimens of the STPH cohort were obtained from Shanghai Tenth People’s Hospital, and other specimens with PD-L1/PD-1 treatment were obtained from Shanghai Chest Hospital. Clinical and pathological characteristics of the patients are shown in Supplementary Table S5. All tumor specimens were approved by the Institutional Review Boards of Shanghai Tenth People’s Hospital (2019-K-9) and Shanghai Chest Hospital (K15-199). Informed consent was obtained from all study participants. For immunohistochemistry, we used vectastain elite ABC kit (PK-6100, Vector) and DAB substrate kit (SK-4100, Vector) according to the manufacturer’s instructions. Briefly, the sections were deparaffinized, rehydrated, incubated with 3% H_2_O_2_ for 20 min at room temperature, and boiled in improved antigen retrieval buffer (36319ES60, YEASEN) for 15 min. After washing 3 times with PBS, the sections were blocked with 3% BSA for 1 h at room temperature and incubated with the primary antibody at 4 °C overnight. The sections were then incubated with the biotinylated secondary antibody for 1 h at room temperature, followed by a chromogenic reaction using vectastain elite ABC kit and DAB substrate kit. Finally, stained sections were counterstained with hematoxylin, dehydrated, and mounted with permount. Images were taken with a Nikon microscopic camera.

The protein levels of MTSS1 and PD-L1 in LUAD specimens were divided into low and high-expression groups according to the intensity of the staining. For T cell analysis, three independent areas with the most abundant infiltration were selected under a microscopic field at 200× magnification, and the numbers of intraepithelial CD8^+^ T cells and GZMB^+^CD8^+^ T cells were counted manually and calculated as a number of cells per field^23^.

### mIHC and image analysis

Tissue sections were blocked with 3% hydrogen peroxide in TBST for 10 min and stained with a multiplex mIHC kit (Panovue, 10217100100). Briefly, the slides were incubated with MTSS1 antibody (CST, 93065) for 60 min, then incubated using the HRP–polymer detection system for 10 min for each step, before visualization using TSA 780 (1:100) for another 10 min. Following this, antigen retrieval was conducted to prepare slides for the next antibody. Using this TSA mIHC method, all samples were stained sequentially with the primary antibodies for PD-L1 (ABCAM, ab213524) visualized with TSA 570 (1:100), CD8A (ABCAM, ab17147) visualized with TSA 480 (1:100), GZMB (CST, 46890) visualized with TSA 690 (1:100), and the KRAS (ABCAM, ab180772) visualized with TSA 620 (1:100). Slides were counterstained with DAPI (Sigma, 1:1000) for nuclei visualization, and subsequently coverslipped using the Hardset mounting media (VectaShield, H-1400).

All tissue sections were imaged using the multispectral imaging system (PerkinElmer, Shanghai Kelin) under the appropriate fluorescent filters for multispectral analysis. A whole slide scan of the multiplex tissue sections produced multispectral fluorescent images visualized in Phenochart (PerkinElmer, Shanghai Kelin) at 200× magnification for further image analysis.

### Tumor–T cell co-culture assays

To analyze the effect of tumor cells on T cell inactivation, 1 × 10^4^ H1975 cells with or without *MTSS1* knockdown were plated into 96-well plates in 100 μL of media, and the adhered H1975 cells were co-cultured with CD3/CD28 (10971, Stemcell) activated Jurkat T cells at a ratio of 1:5 (H1975: Jurkat) for 24 h. Secreted IL-2 level in the medium was measured by a human IL-2 ELISA Kit (BMS221-2, eBioscience) according to the manufacturer’s instructions^18^. To analyze T cell-mediated tumor cell killing, human T cells were activated by culturing human PBMC (PCS-800-011, ATCC) in ImmunoCult-XF T cell expansion medium (10981, Stemcell) with ImmunoCult human CD3/CD28 T cell activator (10971, Stemcell) and IL-2 (10 ng/mL, 78036, Stemcell) for 7 days. Then adhered H1975 cells were co-cultured with activated human T cells at a ratio of 1:10 (H1975: T cells) for 24 h. T cells and cell debris were washed with PBS, and living cells were measured by Cell Counting Kit-8 (HY-K0301, MedChemExpress) according to the manufacturer’s instructions^18^.

### MS analysis

To identify the monoubiquitination site of the PD-L1 protein, 293 T cells were transfected with the indicated constructs. The subsequent immunoprecipitation steps using Flag magnetic beads were the same as those described above. The immunoprecipitates were analyzed with Orbitrap Eclipse (Thermo Scientific, San Jose, CA), and data were analyzed by the National Facility for Protein Science in Shanghai (NFPS), Zhangjiang Lab, China.

### Mouse experiments

All animal studies were conducted according to the guidelines for the care and use of laboratory animals that are approved by the Institutional Biomedical Research Ethics Committee of the Shanghai Institute of Nutrition and Health. The C57BL/6 *Mtss1* KO mice were described as previously^38,49^ and were mated with *Kras*^*LSL-G12D*^ mice (Jackson Laboratory) to generate *Kras*^*LSL-G12D*^; *Mtss1*^*–/–*^ (*KM*^*–/–*^) and *Kras*^*LSL-G12D*^; *Mtss1*^*+/+*^ (*KM*^*+/+*^) mice. Mice of 6–8 weeks old were anesthetized with avertin and then were infected with 5 × 10^6^ PFU Ad-Cre viruses by intranasal instillation^69^. LLC cells (1.25 × 10^6^) with or without *MTSS1* overexpression were injected subcutaneously into the right flanks of 6–8 weeks-old C57BL/6J or BALB/c nude female mice. For combinatory therapy, tumor-bearing C57BL/6J mice were treated with 125 μg of anti-PD1 (RMP1-14, BioXCell, i.p.) or 125 μg rat IgG2a isotype control (2A3, BioXCell, i.p.), and clomipramine (400 μg, i.p.) or saline.

In the model of PBMC-humanized mice, 6–8-weeks-old female NOD.CB17-*Prkdc*^*scid*^*Il2rg*^*tm1*^/Bcgen (NSG) mice were purchased from Beijing Biocytogen Pharmaceuticals Company and NOD.Cg-*Prkdc*^*scid*^*Il2rg*^*tm1Sug*^/JicCrl (NOG) mice were purchased from Zhejiang Vital River Laboratory Animal Technology Company. Human PBMCs were validated by the vendor (Shanghai AoNeng Biotechnology Company) for the establishment of humanized mice. In the experiment of *MTSS1* knockdown, humanized mice were generated by intravenous injection of 8 × 10^6^ PBMCs into NOG mice, and 2.5 × 10^6^ H1975 were injected subcutaneously into the right flanks of mice. In the experiment of combinatory therapy, humanized mice were generated by intravenous injection of 5 × 10^6^ PBMCs into NSG mice, and 0.8 × 10^6^ H1975 were injected subcutaneously into the right flanks of mice. Tumor-bearing humanized mice were treated with 200 μg of anti-PD-L1 (Atezolizumab, HY-P9904, MedChemExpress, i.p.) or human IgG1 isotype control (BE0297, BioXCell, i.p.), and clomipramine (400 μg, i.p.) or saline. Tumor sizes were measured at the indicated times using a caliper and calculated as length × width^2^ × 0.5. Animals were euthanized when tumor diameters reached 1.5 cm, or volumes exceeded 1000 mm^3^, or tumors became ulcerated with the ulcer diameter reaching 1.0 cm.

### CD8^+^ T cell signature score computation

A human LUAD RNAseq dataset (GSE34894^50^) was downloaded from the GEO database. The CD8^+^ T cell enrichment scores of these LUAD samples were analyzed by single-sample gene set enrichment analysis (ssGESA) with a previously published CD8^+^ T cell signature gene set^51^, and then the scores in samples with high or low *MTSS1* mRNA levels were compared by two-tailed unpaired *t*-test.

### scRNA-seq data processing

The murine LUAD scRNA-seq dataset (GSE180964) was obtained from the GEO database. Count matrices were further preprocessed by Seurat R package (v.4.1)^55^. For quality control, cells with ≥ 1000 gene features and genes expressed in ≥ 100 cells were retained for further analysis. Normalized Data function (LogNormalize and 10,000 scale factor parameters) was used for expression data normalizing. Then, 2000 of the most variable genes in the dataset were identified by the FindVariableFeatures function with the vst method. Normalized expression data were scaled and centered by the ScaleData function on the variable genes. RunPCA function was used for principal component analysis on the scaled and centered expression data. The FindNeighbours, FindClusters, and RunUMAP functions were used to cluster the cells and visualize cell clusters, using the first 20 components. Marker genes of each cluster were identified by the FindMarkers function with min.pct = 0.25, respectively. Cell-type preliminary annotations were done with SingleR^70^. Then, cellular marker gene list enrichment analysis (ToppCell Atlas as reference) was performed with marker genes of each cluster by ToppFun^71^ function, and according to the enrichment results, the cell type annotation results were verified and adjusted manually. The resulting Seurat object was used for subsequent analyses.

### Statistics and reproducibility

Statistical analyses were performed using GraphPad Prism 6 software and Microsoft Excel 2010. *P* values were calculated as described in the figure legends. *P* < 0.05 was considered to be statistically significant. Representative results were repeated independently at least two times with similar results.

### Data sources and data availability

The data for analysis of *MTSS1* mRNA and protein expression in human LUAD or NATs were downloaded from the Oncomine database^46^ and the CPTAC data portal^47^, respectively. *MTSS1* mRNA expression and overall survival data were obtained from the cBioPortal database^48^. The dataset used for T cell signature analysis in the ssGESA was downloaded from GEO GSE34894^50^, and the CD8^+^ T cell signature gene set was from a previous publication^51^. The scRNA-seq data for analysis of *Mtss1* mRNA and *Aip4* mRNA expression was downloaded from GEO (GSE180964)^55^. Source data for all the figures in this study are provided with the publication. All other data supporting the findings of this study are available upon reasonable request.

## Supplementary information

Supplementary Figures and Tables
