# Supplementary material for: MTSS1 curtails lung adenocarcinoma immune evasion by promoting AIP4-mediated PD-L1 monoubiquitination and lysosomal degradation
Source: Cell Discov. 2023 Feb 21;9:20. doi: 10.1038/s41421-022-00507-x (PMC9944270; doi:10.1038/s41421-022-00507-x)
Supplement: Supplementary file 1 — Supplementary Figures and Tables [file 41421_2022_507_MOESM1_ESM.pdf]

## Supplementary Figures

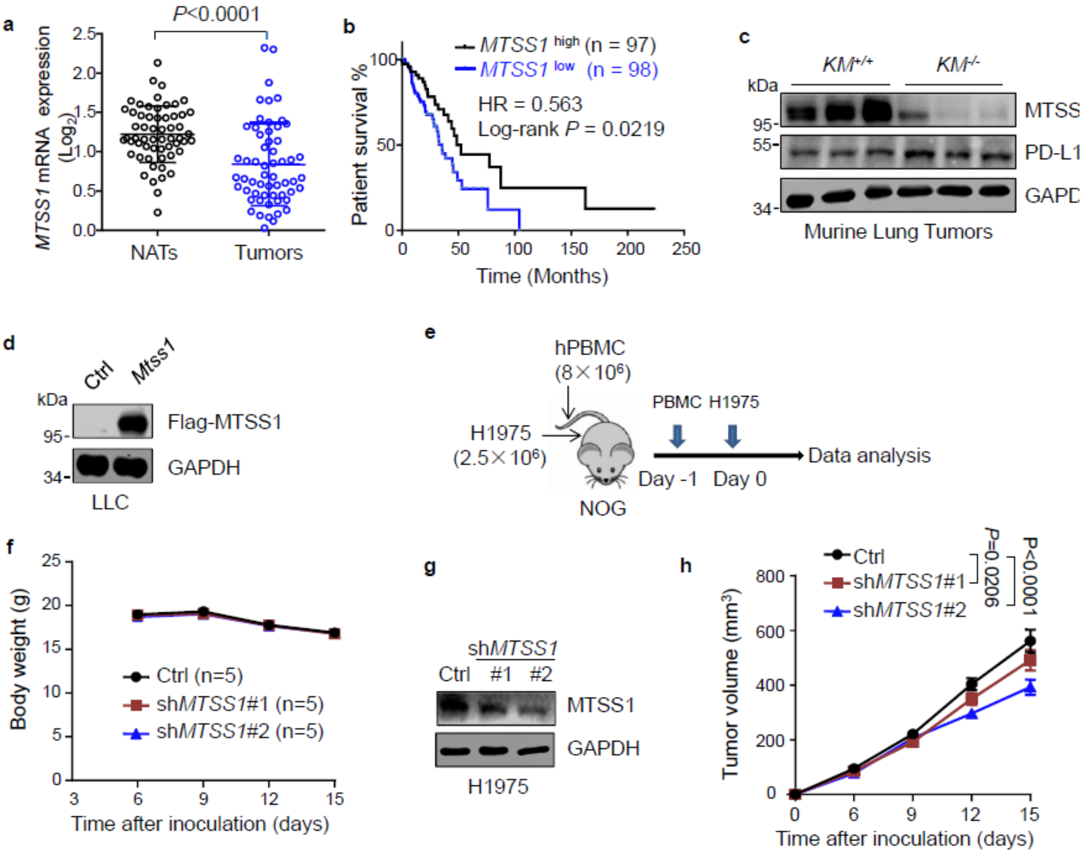


**Supplementary Figure 1. MTSS1 suppresses LUAD.**
**(a)** *MTSS1* mRNA expression in human lung adenocarcinoma (LUAD) samples and normal adjacent tissues (NATs) in the GSE32867 dataset[1]; n = 58 per group.
**(b)** Kaplan-Meier survival curves of LUAD patients in the Cancer Genome Atlas[2] (TCGA) with different *MTSS1* mRNA levels.
**(c)** Immunoblots of lung tumors in $Kras^{LSL\text{-}G12D}$ ($KM^{+/+}$; n = 3) and $Kras^{LSL\text{-}G12D}$; $Mtss1^{-/-}$ ($KM^{-/-}$; n = 3) mice.
**(d)** Immunoblots of LLC with or without *Mtss1* overexpression.
**(e)** A schematic of the procedure to establish H1975 xenograft tumors in PBMC-humanized NOG mice.
**(f)** Body weights of PBMC-humanized NOG mice bearing H1975 xenograft tumors with or without *MTSS1* knockdown.
(**g**) MTSS1 immunoblots in H1975 cells with *MTSS1* knockdown using shRNAs (sh*MTSS1*#1 & #2).
**(h)** Tumor growth of H1975 cells with or without *MTSS1* knockdown in NOG mice (n = 5 per group).
Data are shown as mean ± SD (**a**) or mean ± SEM (**f**, **h**). *P* values were calculated by two-tailed unpaired t test (**a**), log rank test (**b**), or two-way ANOVA (**h**).

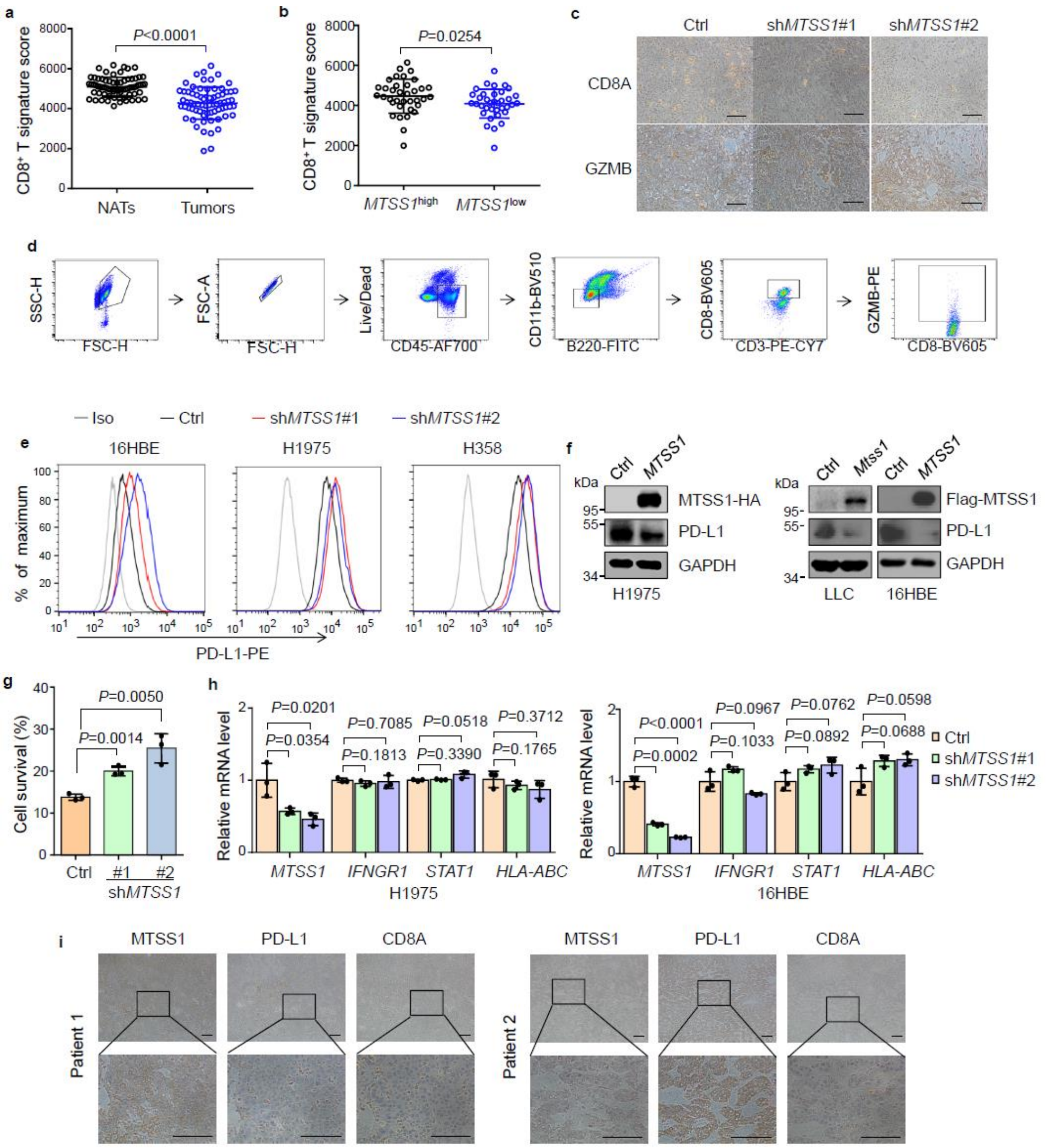


**Supplementary Figure 2. MTSS1 affects CD8$^+$ T cells infiltration and PD-L1 protein expression.**

**(a)** CD8$^+$ T cell signature scores in human LUAD samples and NATs in the GSE34894 dataset[3] (n = 72 per group).

**(b)** CD8$^+$ T cell signature scores in human LUAD samples with different levels of *MTSS1* expression in the GSE34894 dataset[3] (n = 36 per group).

**(c)** Representative images of CD8A and GZMB immunohistochemistry staining of H1975 tumors with or without *MTSS1* knockdown in PBMC-humanized NOG mice.

**(d)** Gating strategy for flow cytometry analysis of the percentages of GZMB$^+$CD8$^+$ T cells in tumors.

**(e)** Representative flow cytometry data of cell surface levels of PD-L1 in the indicated cell lines with *MTSS1* knockdown. The isotype (Iso) was shown as a

negative control.

**(f)** Immunoblots of MTSS1 and PD-L1 in cells with *MTSS1*/*Mtss1* overexpression.

**(g)** Survival of H1975 cells with *MTSS1* knockdown after co-culturing with activated human T cells.

**(h)** qPCR analysis of the indicated genes in H1975 and 16HBE cells with or without *MTSS1* knockdown.

**(i)** Representative images of MTSS1, PD-L1 and CD8A immunohistochemistry staining in human LUAD samples. Scale bars, 100 μm.

Data are shown as mean ± SD (**a**, **b, g, h**). *P* values were calculated by two-tailed unpaired t test (**a**, **b, g, h**).

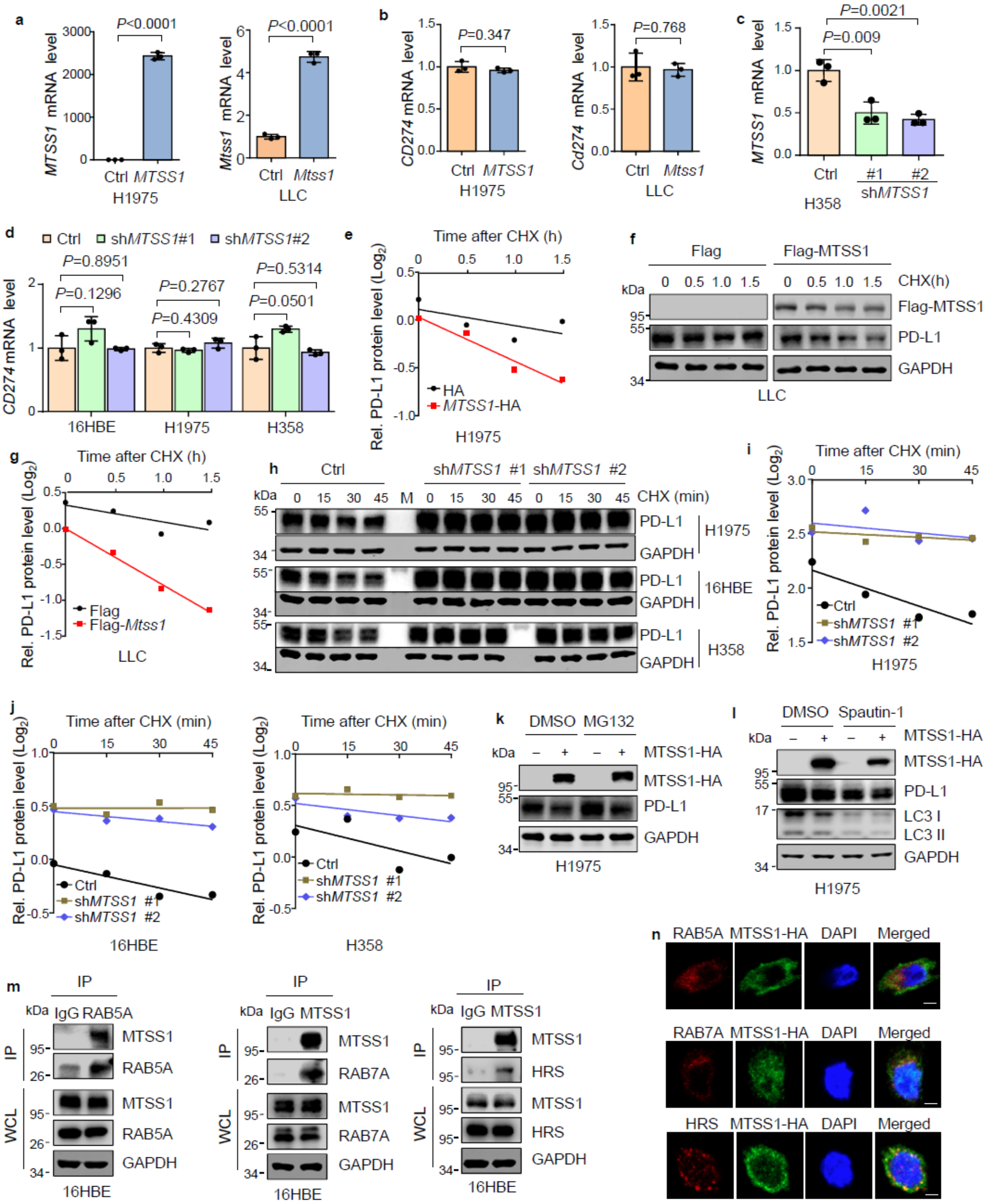


**Supplementary Figure 3. MTSS1 regulates PD-L1 lysosome degradation.**

**(a-d)** qPCR analysis of MTSS1 and CD274 in indicated cell lines with or without *MTSS1* overexpression or knockdown.

**(e)** Protein abundance quantitation of PD-L1 in control and *MTSS1*-overexpressing H1975 cells with treatment of 100 μM cycloheximide (CHX) for the indicated duration.

**(f, g)** Immunoblots (**f**) and quantitation (**g**) of PD-L1 protein in control and *Mtss1*-overexpressing LLC cells with treatment of 100 μM cycloheximide (CHX) for the indicated duration.

**(h-j)** Immunoblots (**h**) and quantitation (**i**, **j**) of PD-L1 protein in 16HBE, H1975

and H358 cells with or without *MTSS1* knockdown after treatment of 300 μM cycloheximide (CHX) for the indicated duration.

**(k, l)** Immunoblots of MTSS1 and PD-L1 in control and *MTSS1*-overexpressing H1975 cells treated with dimethylsulfoxide (DMSO), 25 μM MG132 for 16 h, or 20 μM Spautin-1 for 24 h.

**(m)** Endogenous co-IP analysis of MTSS1 with RAB5A, RAB7A and HRS in 16HBE.

**(n)** Immunofluorescence (IF) analyses for colocalization of MTSS1 with RAB5A RAB7A, or HRS in H1975 cells with *MTSS1* overexpression. Scale bars, 5 μm.

Data are shown as mean ± SD (**a**-**d**). *P* values were calculated by two-tailed unpaired t test (**a**-**d**).

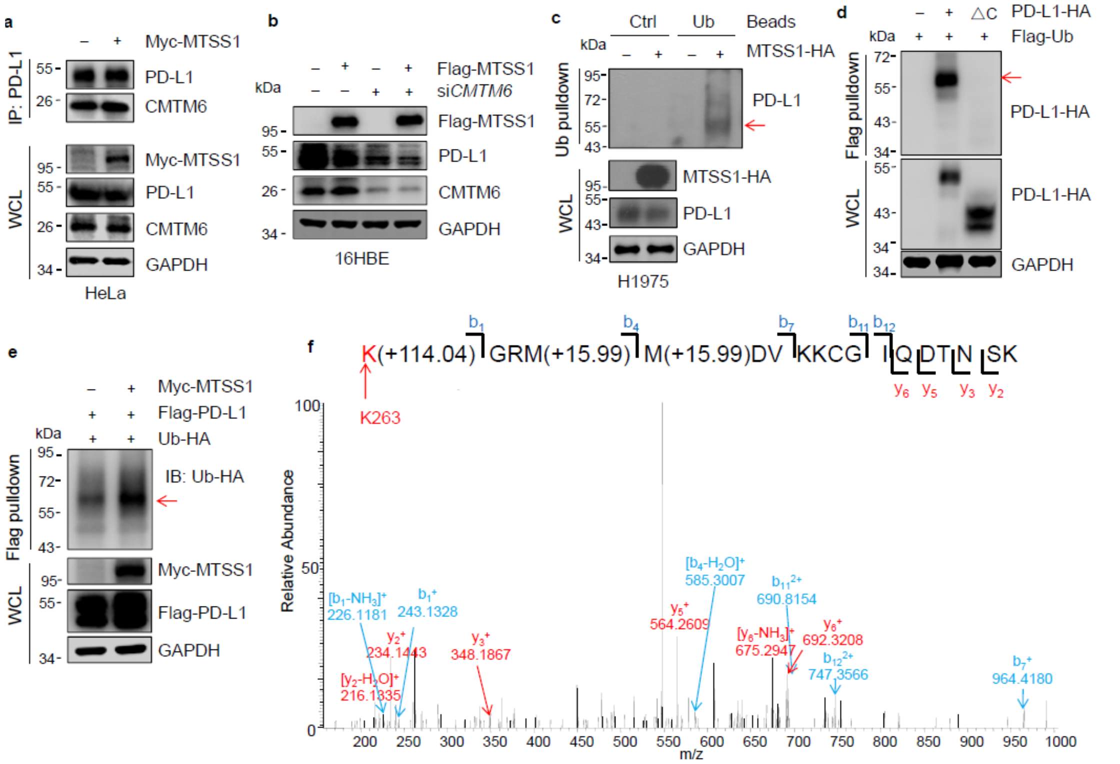


**Supplementary Figure 4. MTSS1 promotes PD-L1 monoubiquitination at K263.**

**(a)** Co-IP of endogenous CMTM6 and PD-L1 in HeLa cells with or without *MTSS1* overexpression.

**(b)** PD-L1 protein levels in 16HBE cells with *CMTM6* knockdown and/or *MTSS1* overexpression.

**(c)** Endogenous PD-L1 ubiquitination analysis in H1975 with or without *MTSS1* overexpression.

**(d)** Ubiquitination analyses of PD-L1 with carboxyl fragment (amino acids 260-290) deletion (△C).

**(e, f)** Mass-spectrometry analysis of PD-L1 ubiquitination. Flag-PD-L1 expressed in 293T cells was pulled down with beads and analyzed by immunoblots (**c**) and mass spectrometry (**d**). Shown in (**d**) is a higher energy collision-induced dissociation (HCD) MS/MS spectrum recorded on the $[M+4H]^{4+}$ ion at m/z 547.0100 of the human PD-L1 peptide KGRMMDVKKCGIQDTNSK harboring one ubiquitination site on lysine and two oxidation sites on methionine. Predicted b- and y-type ions (not including all) are listed above and below the peptide sequence, respectively. Matched ions are labeled in the spectrum and indicate that PD-L1 is ubiquitinated on K263. Arrow points to monoubiquitinated PD-L1 (**c**-**e**).

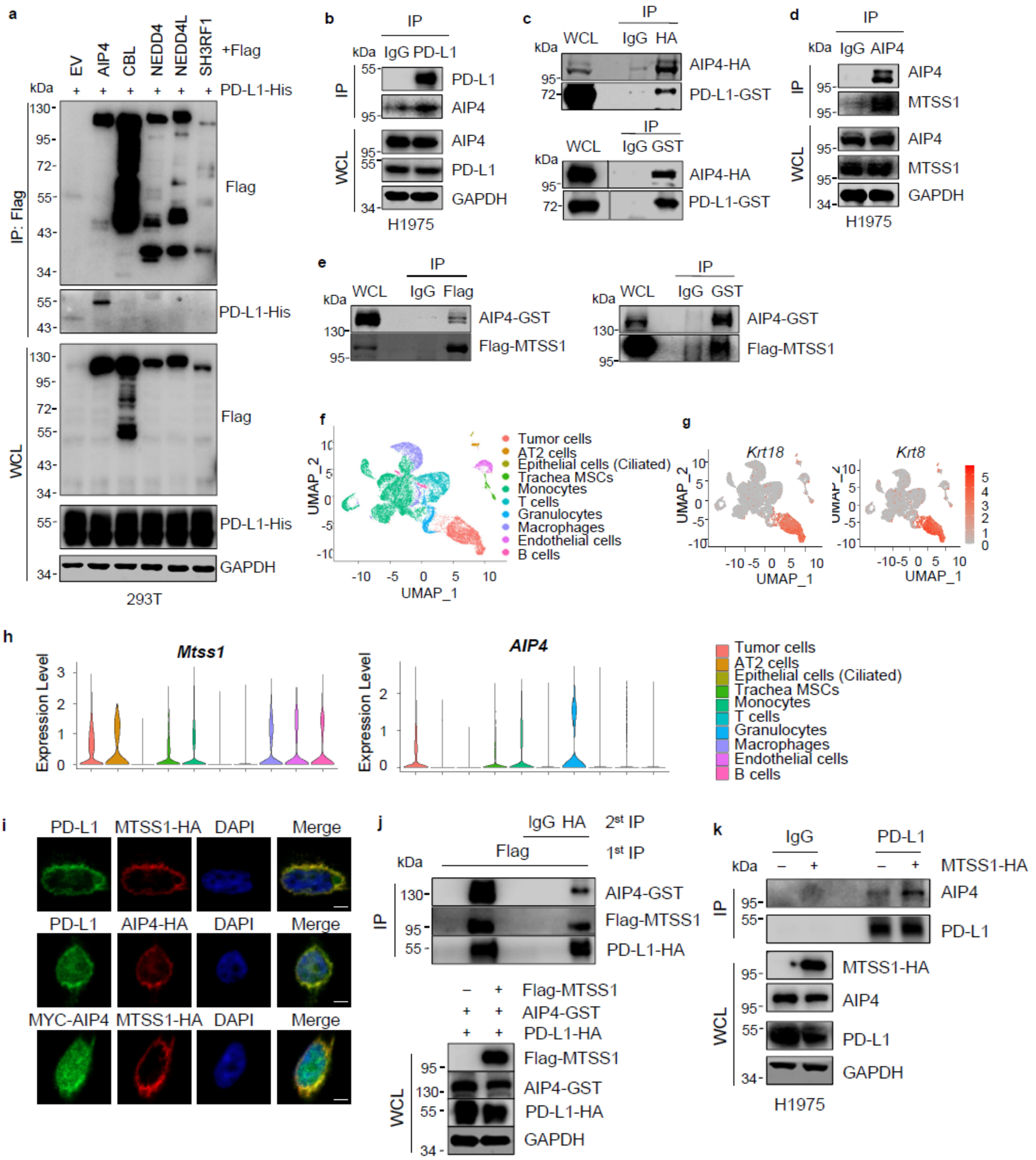


**Supplementary Figure 5. AIP4 interacts with MTSS1 and PD-L1.**

**(a)** Co-IP analyses of PD-L1 with several candidate E3 ligases that have been reported to regulate membrane protein monoubiquitination in 293T.

**(b)** Co-IP assays of endogenous AIP4 with PD-L1 in H1975.

**(c)** Reciprocal co-IP assays of ectopically expressed PD-L1 and AIP4 in 293T.

**(d)** Co-IP assays of endogenous AIP4 with MTSS1 in H1975.

**(e)** Reciprocal co-IP assays of MTSS1 and AIP4 in 293T.

**(f-h)** Analysis of the scRNA data (GSE180964[4]) of 156,000 single cells from 3 murine lung tumors. Shown are Uniform Manifold Approximation and Projection (UMAP) of major cell types (**f**), expression pattern of *Krt18* and *Krt8* in annotated tumor cells (**g**), and expression pattern of *Mtss1* and *Itch* in

different cell types (**h**).

**(i)** IF analyses for colocalization of MTSS1 and PD-L1 (top), AIP4 and PD-L1 (middle), or MTSS1 and AIP4 (bottom) in HeLa cells. Scale bars, 5 μm.

**(j)** Sequential co-IP assay for MTSS1-AIP4-PD-L1 interaction. The assay was performed with an initial IP with Flag beads, and then the precipitated complex was recovered with a secondary IP with anti-HA antibody or IgG in 293T.

**(k)** Co-IP of endogenous AIP4 with PD-L1 in H1975 with or without *MTSS1* overexpression.

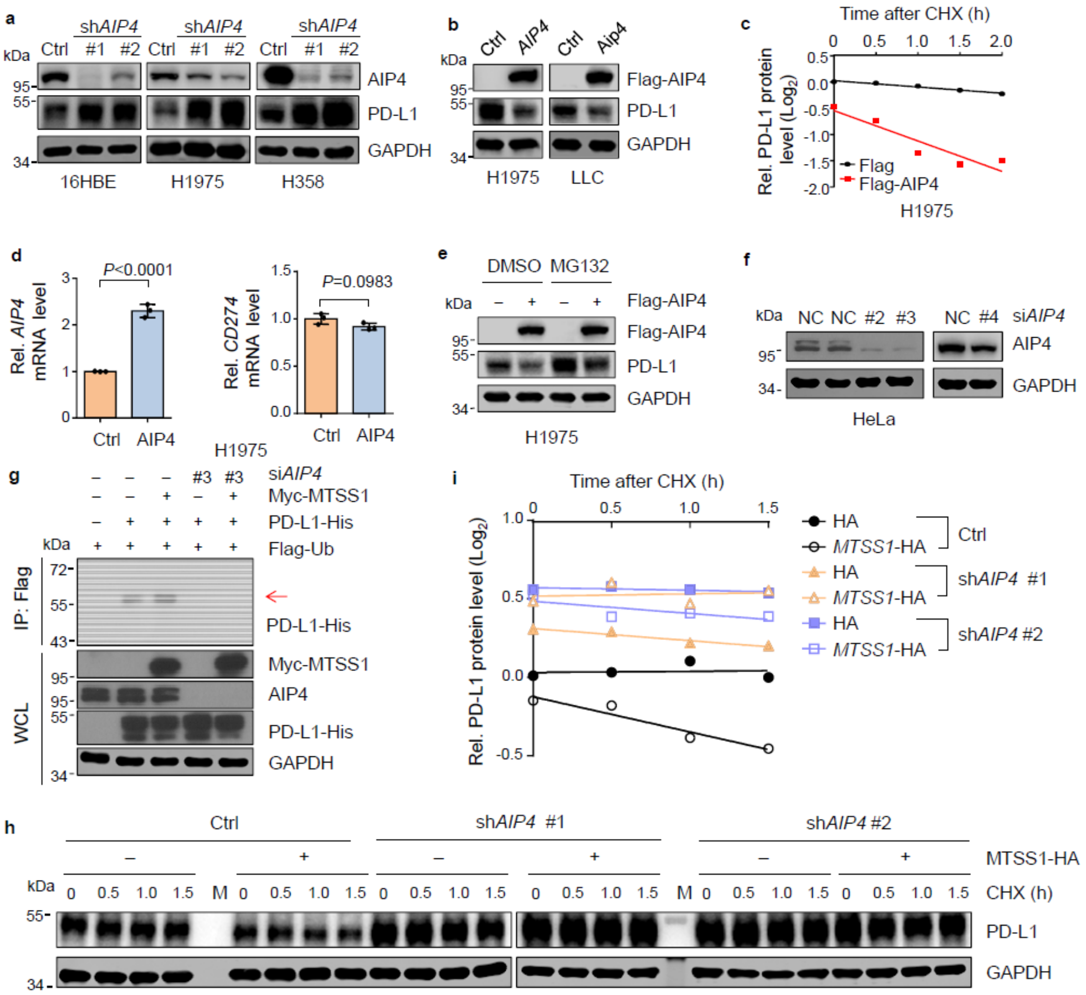


**Supplementary Figure 6. AIP4 regulates PD-L1 protein stability.**

**(a, b)** AIP4 and PD-L1 expression in the indicated cell lines after *AIP4* knockdown (**a**) or overexpression (**b**).

**(c)** PD-L1 abundance quantitation in H1975 with or without *AIP4* overexpression, with treatment of cycloheximide (CHX, 100 μM) for the indicated duration.

**(d)** qPCR analysis of *AIP4* and *PD-L1* mRNA levels in H1975 cells with or without *AIP4* overexpression.

**(e)** AIP4 and PD-L1 immunoblots in H1975 with *AIP4* overexpression after treatment of DMSO or 25 μM MG132 for 16 h.

**(f)** Validation the knockdown efficiency of si*AIP4* by Immunoblots. NC, control.

**(g)** PD-L1 ubiquitination assay in HeLa cells with *AIP4* knockdown and/or *MTSS1* overexpression.

**(h, i)** Immunoblots (**h**) and quantitation (**i**) of PD-L1 protein stability in H1975 with *AIP4* knockdown and/or *MTSS1* overexpression after treatment of 100 μM CHX for the indicated duration.

Data are shown as mean ± SD and *P* values were calculated by two-tailed unpaired t-test (**d**). Arrow points to monoubiquitinated PD-L1 (**g**).

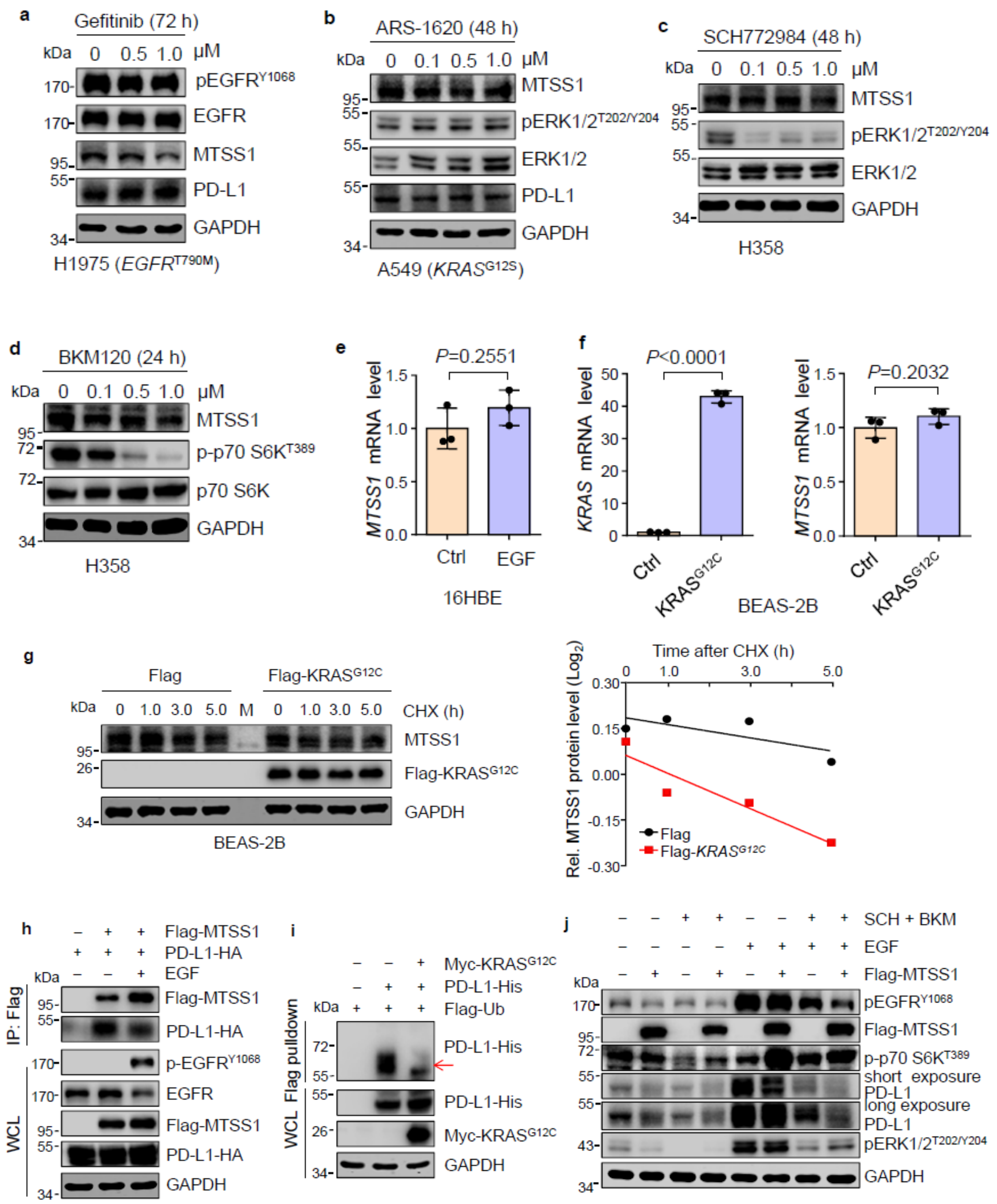


**Supplementary Figure 7. EGFR-KRAS activation inhibits MTSS1 protein expression.**

**(a-d)** Immunoblots of MTSS1, PD-L1 and relevant signaling molecules in H1975 treated with Gefitinib for 72 h (**a**), in A549 treated with ARS-1620 for 48 h in various doses (**b**), in H358 treated with SCH772984 for 48 h in various doses (**c**), and in H358 treated with BKM120 for 24 h in various doses (**d**).

**(e, f)** qPCR analysis of the indicated genes in 16HBE cells treated with 50 ng/ml EGF for 12 h (**e**) and in BEAS-2B cells with or without *KRAS*$^{G12C}$ overexpression (**f**).

**(g)** Immunoblots and quantitation of MTSS1 protein in control and *KRAS*$^{G12C}$-overexpressing BEAS-2B cells with treatment of 100 μM CHX for the indicated

duration.

**(h)** Co-IP analysis of MTSS1 and PD-L1 in 293T after treatment with 50 ng/ml EGF for 15 min.

**(i)** PD-L1 ubiquitination analysis in 293T cells after *KRAS*$^{G12C}$ overexpression. Arrow points to monoubiquitinated PD-L1.

**(j)** Immunoblots of 16HBE treated with EGF (50 ng/ml), SCH772984 (SCH, 1.0 μM) and BKM120 (BKM, 0.5 μM), and/or *MTSS1* overexpression.

Data are shown as mean ± SD and *P* values were calculated by two-tailed unpaired t-test (**e**, **f**).

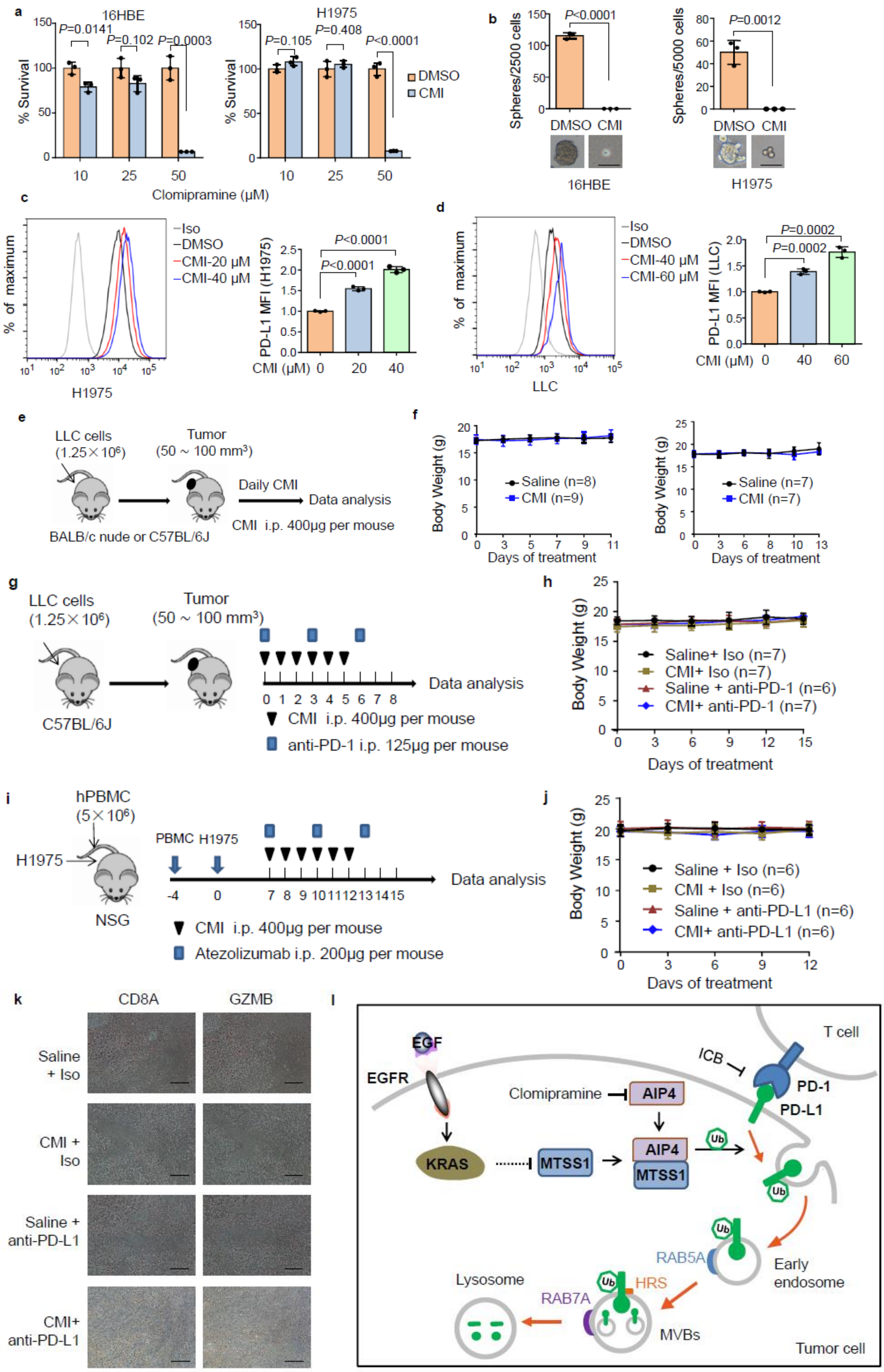


**Supplementary Figure 8. Combining clomipramine with ICB suppresses LUAD development.**

**(a)** Cell survival assays of 16HBE and H1975 cells treated with clomipramine of the indicated concentrations for 48 h.

**(b)** Quantitation and representative images of tumorsphere formation in 16HBE and H1975 cells treated with DMSO or 25 μM clomipramine. Scale bars, 50 μm.

**(c, d)** Flow cytometry analyses of cell surface PD-L1 in H1975 (**c**) and LLC (**d**) treated with clomipramine of the indicated concentrations. MFI, flow cytometry mean fluorescence intensity.

**(e)** A schematic of the procedure for clomipramine treatment of BALB/c nude mice or C57BL/6J mice with LLC xenograft tumors.

**(f)** Body weights of nude mice (left) or C57BL/6J mice (right) treated as in (**e**).

**(g)** A schematic of the procedure for clomipramine and/or anti-PD-1 (RMP1-14) treatment of C57BL/6J mice with LLC xenograft tumors.

**(h)** Body weights of C57BL/6J mice treated as in (**g**).

**(i)** A schematic of the procedure for clomipramine and/or Atezolizumab treatment of PBMC-humanized NSG mice.

**(j)** Body weights of PBMC-humanized NSG mice treated as in (**i**).

**(k)** Representative images of CD8A and GZMB immunohistochemistry staining in LLC tumors treated as in (**i**). Scale bars, 100 μm.

**(l)** A schematic model of the role of MTSS1 to regulate PD-L1 and immune evasion in LUAD.

Data are shown as mean ± SD (**a-d**, **f, h, j**). *P* values were calculated by two-tailed unpaired t test (**a-d**).

## References


1. Selamat, S.A. *et al.* Genome-scale analysis of DNA methylation in lung adenocarcinoma and integration with mRNA expression. *Genome Res* **22**, 1197-1211 (2012).
2. Cancer Genome Atlas Research, N. Comprehensive molecular profiling of lung adenocarcinoma. *Nature* **511**, 543-550 (2014).
3. Derrien, T. *et al.* The GENCODE v7 catalog of human long noncoding RNAs: analysis of their gene structure, evolution, and expression. *Genome Res* **22**, 1775-1789 (2012).
4. Tang, K.H. *et al.* Combined Inhibition of SHP2 and CXCR1/2 Promotes Antitumor T-cell Response in NSCLC. *Cancer Discov* **12**, 47-61 (2022).

**Supplementary Table S1. Reagents used in this study.**

| Reagents | Catalog No. | Vendors |
|---|---|---|
| DMSO | SC-358801 | Santa Cruz |
| MG132 | S2619 | Selleck Chemicals |
| cycloheximide | 1041 | BioVision |
| 3×FLAG peptide | HY-P0319 | MedChemExpress |
| Cell Counting Kit-8 | HY-K0301 | MedChemExpress |
| Chloroquine diphosphate salt solid | C6628 | Sigma |
| Bafilomycin A1 | HY-100558 | MedChemExpress |
| PNGase F | P0708 | New England Biolabs |
| EGF | PHG0311 | Invitrogen |
| Gefitinib | S1025 | Selleck Chemicals |
| WZ4002 | S1173 | Selleck Chemicals |
| ARS-1620 | S8707 | Selleck Chemicals |
| SCH772984 | HY-50846 | MedChemExpress |
| BKM120 | HY-70063 | MedChemExpress |
| Clomipramine hydrochloride | HY-B0457 | MedChemExpress |
| N-Ethylmaleimide | 4259 | Sigma |
| Atezolizumab | HY-P9904 | MedChemExpress |
| Spautin-1 | HY-12990 | MedChemExpress |

**Supplementary Table S2. siRNA and shRNA sequences.**

| ID | Target sequence (5'-3') |
|---|---|
| sh*MTSS1* #1 | TTGTAGGCAACTCGGAATATA |
| sh*MTSS1* #2 | GGCAATTCCAGAAAGTGAATT |
| sh*AIP4* #1 | CCAGTTGGACTCAAGGATTTA |
| sh*AIP4* #2 | GCCTATGTTCGGGACTTCAAA |
| si*AIP4* #2 | GCTACAGCGTAGTCAGCTT |
| si*AIP4* #3 | GCAATGGACCACAGAAATT |
| si*AIP4* #4 | CCCAAGAATCAGAGGTTATAT |
| si*CMTM6* | CTGAGCCACTTAATGCCTA |

**Supplementary Table S3. Antibodies used in this study.**

| Assays | Antibodies | Catalog No. | Vendors |
|---|---|---|---|
| Immunofluorescence | rabbit anti-HA | 3724 | Cell Signalling Technology |
| | mouse anti-HA | H9658 | Sigma |
| | mouse anti-Myc | 2276 | Cell Signalling Technology |
| | mouse anti-Rab5A | 46449 | Cell Signalling Technology |
| | rabbit anti-Rab7 | 9367 | Cell Signalling Technology |
| | rabbit anti-HRS | 15087 | Cell Signalling Technology |
| | rabbit anti-EEA1 | 3288 | Cell Signalling Technology |
| | rabbit anti-LAMP1 | 9091 | Cell Signalling Technology |
| | rabbit anti-PD-L1 | ab213524 | ABCAM |
| | mouse anti-PD-L1 | 14-5983-82 | Thermo Fisher Scientific |
| Immunoblots | rabbit anti-MTSS1 | 93065 | Cell Signalling Technology |
| | rabbit anti-PD-L1 | 13684 | Cell Signalling Technology |
| | mouse anti-PD-L1 | 29122 | Cell Signalling Technology |
| | rabbit anti-PD-L1 | ab213480 | ABCAM |
| | rabbit anti-AIP4 | 12117 | Cell Signalling Technology |
| | mouse anti-AIP4 | sc-28367 | Santa Cruz |
| | rabbit anti-EGFR | sc-03 | Santa Cruz |
| | rabbit anti-Phospho-EGFR | 3777 | Cell Signalling Technology |
| | rabbit anti-Erk1/2 | 9102 | Cell Signalling Technology |
| | rabbit anti-Phospho-Erk1/2 | 4370 | Cell Signalling Technology |
| | rabbit anti-p70 | 2708 | Cell Signalling Technology |

| | | | |
|---|---|---|---|
| | rabbit anti-Phospho-p70 | 2708 | Cell Signalling Technology |
| | mouse anti-Rab5A | 46449 | Cell Signalling Technology |
| | rabbit anti-Rab7 | 9367 | Cell Signalling Technology |
| | rabbit anti-HRS | 15087 | Cell Signalling Technology |
| | rabbit anti-MTSS1 | 4385 | Cell Signalling Technology |
| | rabbit anti-LC3 I /LC3 II | NB100-2220 | Novus Biologicals |
| | rabbit anti-CMTM6 | 90329 | Cell Signalling Technology |
| | rabbit anti-HA | 3724 | Cell Signalling Technology |
| | mouse anti-HA | H9658 | Sigma |
| | mouse anti-Flag | F1804 | Sigma |
| | rabbit anti-GST | 2625 | Cell Signalling Technology |
| | rabbit anti-His | 12698 | Cell Signalling Technology |
| | mouse anti-Myc | 2276 | Cell Signalling Technology |
| | rabbit anti-GAPDH | G9545 | Sigma |
| | IRDye 800CW donkey-anti-rabbit secondary antibody | 926-32213 | LICOR |
| | peroxidase-conjugated goat anti-mouse secondary antibody | 401215 | Merck/millipore |
| | peroxidase-conjugated goat anti-rabbit secondary antibody | 401315 | Merck/millipore |
| | Rabbit anti-PD-L1 | 13684 | Cell Signalling Technology |
| | mouse anti-AIP4 | sc-28367 | Santa Cruz |
| | mouse anti-Rab5A | 46449 | Cell Signalling Technology |

| | | | |
|---|---|---|---|
| Immunoprecipitation | rabbit anti-GST | 2625 | Cell Signalling Technology |
| | mouse anti-Flag | F1804 | Sigma |
| | mouse anti-HA | H9658 | Sigma |
| | anti-Flag magnetic beads | M8823 | Sigma |
| | Signal-Seeker Ubiquitin Enrichment Kit | BK161 | Cytoskeleton |
| | rabbit IgG isotype control | 3900 | Cell Signalling Technology |
| | mouse IgG isotype control | sc-2025 | Santa Cruz |
| Immunohistochemistry | Rabbit anti-MTSS1 | 93065 | Cell Signalling Technology |
| | rabbit anti-PD-L1 | ab213524 | ABCAM |
| | mouse anti-CD8A alpha | ab17147 | ABCAM |
| | rabbit anti-Granzyme B | 46890 | Cell Signalling Technology |
| | rabbit anti-KRAS | ab180772 | ABCAM |
| | biotinylated goat anti-mouse IgG antibody | BA-9200 | Vector |
| | biotinylated goat anti-rabbit IgG antibody | 111-065-144 | JACKSON |
| Flow cytometry | PE mouse IgG2b, κ isotype control | 400314 | Biolegend |
| | PE anti-human PD-L1 | 329706 | Biolegend |
| | Alexa Fluor® 700 anti-mouse CD45 | 103127 | Biolegend |
| | PE/Cy7 anti-mouse CD3 | 100319 | Biolegend |
| | Brilliant Violet 605™ anti-mouse CD8 | 100743 | Biolegend |
| | Brilliant Violet 510™ anti-mouse CD11B | 101245 | Biolegend |

| | | | |
|---|---|---|---|
| | FITC anti-mouse B220 | 103205 | Biolegend |
| | PE anti-mouse granzyme B | 12-8898-80 | Invitrogen |
| | Fixable Viability Dye eFluor™ 780 | 65-0865-14 | Invitrogen |
| | CD16/CD32 | 553142 | BD Biosciences |

**Supplementary Table S4. Primers for qRT-PCR.**

| Gene | | Sequence (5'-3') |
|---|---|---|
| *MTSS1* | Forward | GCTTCGGACAACAGTAGTAGCA |
| | Reverse | CAGATCCAATCTCCCTGGTC |
| *AIP4* | Forward | CATGGGATAGACCAGAACCTCT |
| | Reverse | ACATAATAAATACGTCCCATGTTGTC |
| *PD-L1* | Forward | GGCATTTGCTGAACGCAT |
| | Reverse | CAATTAGTGCAGCCAGGT |
| *HLA-ABC* | Forward | CCTACGACGGCAAGGATTAC |
| | Reverse | TGCCAGGTCAGTGTGATCTC |
| *IFNGR1* | Forward | AGTGCTTAGCCTGGTATTCATCTG |
| | Reverse | GGCTGGTATGACGTGATGAGTG |
| *STAT1* | Forward | ATCAGGCTCAGTCGGGGAATA |
| | Reverse | TGGTCTCGTGTTCTCTGTTCT |
| *GAPDH* | Forward | GAAGGTGAAGGTCGGAGTC |
| | Reverse | GAAGATGGTGATGGGATTTC |
| *Mtss1* | Forward | CTTCATAAACAAAGCCGGAAA |
| | Reverse | GCTGCTGCTACTACTGTTGTCC |
| *Aip4* | Forward | CAAGCTTTCTTTGAGGGCTTTA |
| | Reverse | GCATCCCACACAGAAGAACC |
| *Pd-l1* | Forward | CCATCCTGTTGTTCCTCATTG |
| | Reverse | TCCACATCTAGCATTCTCACTTG |
| *β-actin* | Forward | GGCTGTATTCCCCTCCATCG |
| | Reverse | CCAGTTGGTAACAATGCCATGT |

**Supplementary Table S5. Clinical and pathological characteristics of LUAD patients.**

| | No. (%) of patients (n=15) |
|---|---|
| **Age (Averge Age= 63.5)** | |
| <= 64 | 7 (46.7) |
| > 64 | 8 (53.3) |
| **Gender** | |
| Male | 11 (73.3) |
| Female | 4 (26.6) |
| **Stage** | |
| I - II | 10 (66.6) |
| III - IV | 5 (33.3) |
| **T stage** | |
| T1 - T2 | 5 (33.3) |
| T3 - T4 | 10 (66.6) |
| **N stage** | |
| N0 | 2 (13.3) |
| N1 - N3 | 13 (86.7) |
| **M stage** | |
| M0 | 1 (6.7) |
| M1 | 14 (93.3) |
| **Response to ICB therapy** | |
| PD | 1 (6.7) |
| SD | 8 (53.3) |
| PR | 6 (40) |
| **Gene mutation** | |
| KRAS | 7 (46.7) |
| EGFR 19DEL | 2 (13.3) |
| No | 2 (13.3) |
| ND | 4 (26.6) |
| | |
| ND: No data | |
